# Application of Nanoparticles in Plant In Vitro Culture for Micropropagation and Secondary Metabolite Production: A Review

**DOI:** 10.3390/plants15132071

**Published:** 2026-07-03

**Authors:** Natalia A. Semenova, Dmitry A. Zakharov, Dmitry A. Serov, Sergey V. Gudkov, Alexey S. Dorokhov, Andrey Yu. Izmailov

**Affiliations:** 1 Prokhorov General Physics Institute, Russian Academy of Sciences, Vavilov Str. 38, 119991 Moscow, Russia; natalia.86@inbox.ru (N.A.S.); zaharov121221@mail.ru (D.A.Z.); dmitriy_serov_91@mail.ru (D.A.S.); 2 Federal Scientific Agroengineering Center VIM, 109428 Moscow, Russia; dorokhov.vim@yadex.ru (A.S.D.); vim@vim.ru (A.Y.I.); 3 Department of Fundamental Sciences, Bauman Moscow State Technical University, 5 2nd Baumanskaya Str., 105005 Moscow, Russia

**Keywords:** nanoparticles, plant tissue culture, micropropagation, explant sterilization, shoot multiplication, in vitro rooting, elicitation, secondary metabolites, hairy root culture

## Abstract

This review summarizes recent studies on the use of nanoparticles (NPs) in plant in vitro clonal micropropagation and secondary metabolite production. The analyzed applications include culture initiation, shoot multiplication, rooting, acclimatization, callus culture, and hairy root culture. Because NPs’ effects are strongly endpoint-dependent, their effectiveness is evaluated separately for micropropagation endpoints, including contamination control, explant survival, multiplication rate, shoot and root development, and plantlet quality, and for metabolite-production endpoints, including biomass accumulation, target metabolite concentration, and total metabolite yield. Based on the quantitative analysis of published data, the most frequently beneficial NP size range was 20–60 nm, whereas effective concentrations, combining a positive effect on plant growth and the synthesis of secondary metabolites, were mainly within 1–120 mg L^−1^. For the most extensively studied NPs, the corresponding indicative concentration ranges were 15–45 mg L^−1^ for 25–40 nm Ag-NPs, 75–200 mg L^−1^ for 15–45 nm ZnO-NPs, 120–150 mg L^−1^ for 50–80 nm Se-NPs, and 90–145 mg L^−1^ for 54–55 nm Si-based NPs. Ag- and carbon-based nanomaterials showed relatively strong overall responses in micropropagation datasets, whereas Ag- and Se-NPs were often associated with enhanced target metabolite accumulation. NP responses depend on particle composition, synthesis method, surface properties, dose, culture system, species, genotype, and may involve phytotoxic or residue-related risks.

## 1. Introduction

The growing demand for food, high-quality planting material, and biologically active plant-derived compounds is driving the rapid development of agricultural biotechnology. Population growth, climate change, the reduction of arable lands, and the lack of applying sustainable and resource-efficient technologies stimulate the development of controlled production systems that are less dependent on seasonality and environment conditions [1,2,3,4,5,6]. In this context, plant in vitro culture should be considered as an advanced technology. The conceptual basis for the possibility of culturing plant cells is associated with the work of the Austrian botanist and plant physiologist Gottlieb Haberlandt, who conducted a series of experiments and formulated perspectives of totipotency in plant cell culture [7], which were successfully implemented half a century later [8,9]. Plant tissue culture has greatly expanded the possibilities for regenerating plants from highly differentiated, structurally and functionally specialized cells of leaves, roots, stems, floral organs, and endosperm.

At present, plant tissue culture methods make it possible to address key challenges in the commercial clonal micropropagation and sanitation of plants, plant breeding and genetic modification, the conservation of endangered species and rare cultivars, as well as the production of secondary metabolites for applications across various sectors of industry [10]. In this context, considerable progress has been observed in the development of materials and technologies that improve the efficiency of in vitro processes and increase production output. In particular, the application of nanoparticles (NPs) with sizes ranging from 1 to 100 nm is becoming increasingly important because of their unique properties, including large surface area, increased surface energy, and quantum size effects. In plant in vitro culture systems, NPs can perform several different and sometimes interrelated functions. They may act as antimicrobial agents during culture initiation, sources of nutrients or released ions, modulators of oxidative and abiotic stress responses, factors involved in reactive oxygen species (ROS)-mediated signaling, elicitors of secondary metabolism, or, at excessive concentrations, toxic agents that disrupt growth, morphogenesis, or genetic stability [11,12]. ROS generated under the influence of NPs may serve as triggers for the induction of secondary metabolism in plants [13]. Secondary metabolites may function as phytoalexins or phytoanticipins, providing protection against biotic and abiotic stresses [14] and may also act as antioxidants that neutralize ROS [15,16,17,18].

NP uptake by plants may occur either before transfer into sterile culture through immersion in a NP-containing solution or directly in plant tissue culture through artificial nutrient media [19,20]. Depending on the conditions, NPs may adsorb onto the cell wall surface, penetrate through existing pores or damaged regions, participate in endocytosis-like processes, or partially dissolve with the release of ions that are subsequently transported through ion channels or carriers [21]. Owing to their higher surface-area-to-mass ratio compared with conventional bulk metallic forms, NPs exhibit enhanced reactivity in interactions with the surrounding environment [21], which may promote the formation of complexes with membrane transporters or root exudates before uptake. NPs may also aggregate or agglomerate, dissolve with ion release, interact with agar, salts, organic compounds, plant growth regulators, and exudates, or adsorb onto plant surfaces [22,23,24].

Therefore, the unique properties of nanomaterials are increasingly being used not only in agriculture [14,25] but also in agrobiotechnology to suppress bacterial and fungal infections, stimulate shoot and callus growth, enhance pigment synthesis [26,27,28], and serve as elicitors for inducing abiotic stress that stimulates secondary metabolite production [29,30]. These metabolites may be applicable in the cosmetic, food, and pharmaceutical industries [22,31,32,33]. Innovative approaches such as plant nanobiotechnology have the potential to enhance plant tolerance to stress factors, including salinity and water deficit [14,25,34,35,36,37,38]. These advances create opportunities for the transition toward sustainable, highly productive, and environmentally safe agricultural practices, as well as for the production of high-quality and safe food products, including functional foods.

It is well established that the effectiveness of NPs is determined by their size, charge, and synthesis method [14]. NPs can be produced using physical, chemical, or biological methods. Physical methods, such as milling, laser ablation, inert gas condensation, and magnetron sputtering, allow the production of highly pure particles with controlled size and morphology [39,40,41], whereas chemical methods, including solvothermal, sonochemical, and microwave-assisted synthesis, allow to obtain the desired particle size, shape, and surface properties [42,43,44,45,46]. Green synthesis is often considered a more environmentally friendly approach and may improve biocompatibility; however, precise control of particle size, shape, and surface chemistry may be more difficult than in chemical or physical synthesis [47,48,49,50]. These differences are important because the effects of NPs in vitro may depend not only on chemical composition but also on particle size distribution, surface coating, charge, stability, and the presence of residual synthesis components.

Nanobiotechnology is a relatively new field that offers solutions to various biotechnological challenges through the use of methods and tools operating at the nanoscale. Therefore, existing studies on the application of NPs in plant in vitro culture are highly heterogeneous. They differ in NP type, particle size, synthesis method, surface charge, coating, concentration, exposure duration, nutrient medium composition, plant species, genotype, explant type, culture stage, and evaluated endpoint. This heterogeneity complicates direct comparison of results and prevents the identification of universal and effective NP types or concentrations. Therefore, a stage-specific and mechanism-oriented analysis of the data is needed to determine which NP properties and application conditions are associated with beneficial effects during culture initiation, shoot multiplication, rooting, acclimatization, callus growth, and hairy root culture, as well as which conditions increase the probability of adverse outcomes.

**Figure 1:**
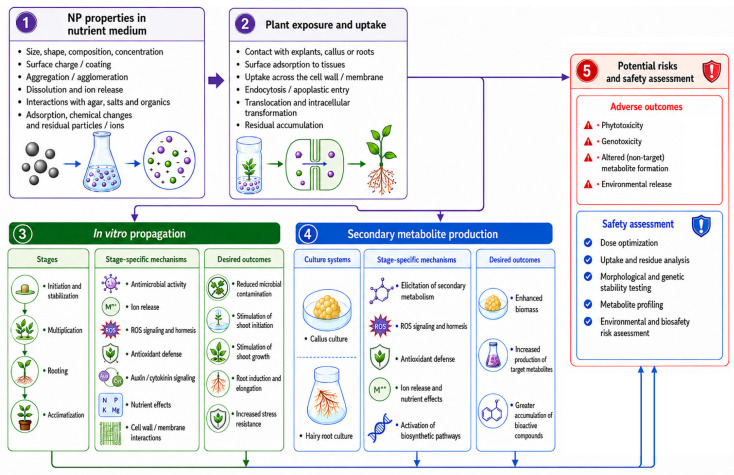
Conceptual framework for the application of nanoparticles in plant in vitro culture: NP properties and transformation in the culture medium, exposure of plant tissues and uptake, stage-specific mechanisms of action, desired effects in micropropagation and secondary metabolite production, and potential risks and safety assessment.

The present review summarizes and systematizes current data on the application of NPs in plant in vitro culture, with an emphasis on clonal micropropagation and the production of target metabolites in callus and hairy root cultures. Cell suspension cultures are not analyzed in detail because they represent a separate liquid and bioreactor-based cultivation platform, in which NP behavior is additionally determined by hydrodynamics, aeration, sedimentation, aggregation, shear stress, and mass-transfer conditions. These systems are highly important for industrial metabolite production and elicitation studies; however, they require separate analysis. Accordingly, the present review focuses on semi-solid and organ-/tissue-based in vitro systems and considers NP effects in relation to culture stage, biological endpoints, and known limitations (Figure 1).

## 2. Application of NPs in Clonal Plant Micropropagation

Clonal micropropagation of plants is a method of reproduction in which plants are cultivated from small parts (explants) of a maternal plant under sterile conditions. Explants may consist of cells, tissues, or even plant organs. This method enables the rapid production of a large number of genetically identical plants (clones) and is widely used in agriculture, horticulture, and forestry for the efficient reproduction and conservation of plant species [51,52]. Over the past decade, the popularity of micropropagation has increased substantially. In 2023–2024, the average annual growth rate of the financial segment of the clonal propagation market reached 10% [53]. The increasing popularity of this method is attributed to the high demand for healthy planting material, the need to conserve rare and endangered plant species [20], and the method’s broad applications in breeding, high multiplication rates, and independence from weather and seasonal conditions [21,54].

Recent studies indicate growing interest in the application of various NPs in in vitro tissue culture [13,22,23,55]. NPs are utilized at all stages of clonal micropropagation for various purposes: explant sterilization, plant growth stimulation [13], enhancement of multiplication rates [56,57], stimulation of root initiation, stimulation of secondary metabolite synthesis [58,59], and improved adaptation of plants to non-sterile environmental conditions [60]. Most research on the use of nanoparticles in clonal micropropagation focuses on explant sterilization before their introduction into in vitro culture, explant growth initiation, and the multiplication stage.

### 2.1. Application of NPs in Culture Initiation and Stabilization Stage In Vitro

The main challenges encountered in biotechnology during the initiation of plant tissue culture are associated with the difficulty of eliminating microbial contamination and the toxicity of many sterilizing agents used both for plant explants and for operators. Plant explants are commonly sterilized using solutions containing active chlorine compounds, including sodium and calcium hypochlorites and chloramine, as well as mercuric chloride, hydrogen peroxide, ethanol, diocide, antibiotics, and other substances [61,62]. These methods, applied either separately or in combination, can disinfect 15–90% of explants depending on the plant species; however, explant survival often remains low [63]. Taken together, these difficulties increase the labor intensity and cost of the stage of introduction into sterile culture.

The use of NPs at carefully selected concentrations may be considered a modern and promising alternative to conventional disinfectants, since they may exhibit lower phytotoxicity and reduced risks to human health. Recently, silver nitrate, a more expensive but less toxic alternative to traditional agents, has been used for explant sterilization and demonstrated antibacterial efficacy during the cultivation of lingonberry (*Vaccinium vitis-idaea* L.) and *Vaccinium praestans* Lamb., ensuring explant survival of up to 72% and 96%, respectively [51]. Ag-NPs possess bactericidal properties at substantially lower concentrations, which makes them a more attractive and promising option as sterilizing agents for the establishment of axenic cultures [64]. In addition, reducing NP size increases their bactericidal effectiveness [63]. Compared with AgNO_3_, Ag-NPs did not induce oxidative damage in the tissues of *Colobanthus quitensis* (Kunth) Bartl. and *Deschampsia antarctica* É.Desv., although no bactericidal effect was observed in these species (Table 1). This was probably associated with the relatively large particle size of 50 nm, whereas optimal sizes are usually within the range of 1–35 nm [63]. The application of AgO-NPs by immersing rhizomes of *Kaempferia nigrifolia* Boonma & Saensouk for 60 min before introduction into tissue culture effectively eliminated contamination without negatively affecting explant survival (100%) [19]. The use of CuO-NPs is also promising, since concentrations of 0.1–5 mg L^−1^ have been shown to exert a sterilizing effect against various phytopathogens without reducing explant viability [65].

It is well established that Se-NPs have sterilizing effects and exhibit antimicrobial, antiviral, and antifungal activities [70,71]. In addition, they stimulate growth, as confirmed by experiments on the cultivation of explants of *Momordica charantia* L. and *Nicotiana tabacum* L. [66,67].

At the stage of tissue culture initiation, graphene oxide (GO) was used to stimulate seedling development while maintaining genetic stability [68]. In addition, multi-walled carbon nanotubes stimulated vigorous shoot growth but induced premature root formation and callus formation [29]. It was also shown that α-Fe_2_O_3_-NPs increased regeneration frequency and shoot growth while simultaneously stimulating root formation [69].

The application of NPs at the stage of tissue culture initiation for explant sterilization and the induction of primary plant growth remains insufficiently studied. An important task is to optimize NP size, concentration, and exposure duration for the sterilization of different plant species. At properly selected concentrations, nanoparticles synthesized by chemical and physical methods may be as effective as particles obtained through biological synthesis, since they do not exert pronounced negative effects on plants. However, the multifunctionality of NPs and the possible occurrence of undesirable effects during micropropagation, such as callus formation and premature root initiation, should be taken into account.

Various metallic NPs with proven antibacterial activity, including silver, copper, zinc, gold, and iron nanoparticles, as well as promising carbon nanomaterials, may be used for explant sterilization [72,73]. It is advisable to test iron oxide nanoparticles (IONPs), which are characterized by pronounced antimicrobial properties and high biocompatibility [74]. Titanium dioxide nanoparticles (TiO_2_-NPs), known for their high biocompatibility and antimicrobial activity, particularly in amorphous form and when doped with inorganic compounds, are also considered promising [75]. For explant sterilization, nanocomposites based on borosiloxane polymers in combination with aluminum oxide nanoparticles (Al_2_O_3_-NPs) may also be used, since they are capable of capturing bacterial cells adhered to their surfaces [76]. This area of NP application is relatively new and has considerable potential for further development.

### 2.2. Application of NPs in Shoot Multiplication Stage (Serial Subcultures)

In vitro shoot multiplication is a critically important stage that involves the controlled initiation and growth of shoots from sterile explants. The aim of this phase is the rapid production of shoots from a single initial explant, which is quantitatively assessed by the multiplication factor (MF), i.e., the number of shoots per explant. This is achieved by enriching the nutrient medium with phytohormones, that trigger the biosynthesis of endogenous auxin [77]: auxins (indole-3-acetic acid (IAA), 1-Naphthaleneacetic acid (NAA), Indole-3-butyric acid (IBA) and cytokinins (6-benzylaminopurine (BA), 6-furfurylaminopurine, or kinetin (Kn), thidiazuron (TDZ), and others)). These compounds are used to stimulate cell division and initiate the development of lateral buds. Microshoots obtained from an explant are transferred to fresh nutrient medium every 3–8 weeks, depending on the plant species. Each cultivation cycle results in a multiplicative increase in shoot number, and once a sufficient amount of material has been obtained, the shoots are prepared for rooting and subsequent acclimatization to non-sterile conditions. During micropropagation, some plant species, especially those characterized by a high MF and the ability to rapidly accumulate biomass, are also cultivated for the production of secondary metabolites [78,79].

Excessive concentrations of hormones may cause developmental abnormalities, such as shortened shoots and tissue vitrification [80]. Therefore, reducing cytokinin doses and/or replacing them with additives that promote the formation of new shoots is of current interest. In this context, the application of NPs is promising for in vitro plant proliferation. It is well established that NP type and size substantially affect the growth and development parameters of microplants. For example, the application of ZnO-NPs with an average size of 25 nm at different concentrations during shoot multiplication of *Chrysanthemum* × *morifolium* resulted in the highest MF values of 6.5 and 10.3 after treatment with 100 and 500 mg L^−1^, respectively [20] (Table 2). However, in *Maerua oblongifolia* Forssk. (A. Rich.), the addition of ZnO-NPs with a size of 10–50 nm, obtained by green synthesis, increased the MF more than twofold at a concentration of 5 mg L^−1^, while the shoots showed improved development and a higher content of photosynthetic pigments [81].

For the micropropagation of *Reseda lutea* L., the most effective treatment involved ZnO-NPs with a size of 26–98 nm, also obtained by green synthesis using *Convolvulus arvensis* L., at concentrations of 15 and 30 mg L^−1^. This resulted in MF increases of 75% and 50%, respectively, from 8 to 12–14 shoots per explant [82]. Notably, treatment with 15 mg L^−1^ also increased protein accumulation and total chlorophyll content. In contrast, the higher concentration of 60 mg L^−1^ was subtoxic and produced no positive effects. The ability of ZnO-NPs to stimulate plant growth, along with their cytotoxicity in vitro, similarly to de novo application [83], exhibits substantial variability (by 1–2 orders of magnitude). This depends on the physicochemical characteristics of the NPs, the environmental conditions, and species-specific traits of the plants. Therefore, the identification of an effective concentration must be carried out over a very broad range for each specific case, which, to date, has prevented the standardization of using this type of NP.

In the leaf segment culture of *Ajuga multiflora* Bunge, the addition of CuO-NPs smaller than 50 nm was also used to enhance the MF [84]. The highest MF, expressed as the number of adventitious and axillary shoots (29 and 22 shoots per explant, respectively), was achieved with the addition of 5 mg L^−1^ CuO-NPs. At higher concentrations, the MF gradually decreased, reaching 1.1 at 40 mg L^−1^. The maximum FW was observed on the medium containing 10 mg L^−1^ CuO-NPs. It was established that low concentrations of CuO-NPs act as elicitors, stimulating sterol synthesis, whereas high concentrations above 20 mg L^−1^ induce oxidative stress and exhibit phytotoxicity [84].

In another study, the effects of two types of NPs, Ag-NPs and Cu-NPs, on the in vitro propagation of *Origanum petraeum* Danin and the composition of secondary metabolites in microshoots were investigated. Among all tested concentrations (0, 25, 50, 100, and 150 mg L^−1^), the optimal effect was observed with Ag-NPs at a concentration of 100 mg L^−1^ [85]. These NPs stimulated microshoot formation, with an MF of up to 12, and enhanced shoot growth. Transmission electron microscopy confirmed nanoparticle uptake and translocation: Ag-NPs were detected in root cells as particles ≤ 24.63 nm, whereas Cu-NPs were detected in leaves as aggregates approximately 50 nm in size. For stimulation of secondary metabolite synthesis, the best results were obtained with 50 mg L^−1^ Cu-NPs, which increased monoterpene content, and 50 mg L^−1^ Ag-NPs, which increased sesquiterpene content.

The effect of Ag-NPs on clonal propagation of apricot shoots (*Prunus armeniaca* L.) substantially depended on the cultivation system [86]. In semi-solid medium, Ag-NPs 60–80 nm in size inhibited shoot growth, whereas in temporary immersion systems (TIS), they improved proliferation and biomass accumulation. The optimal results were achieved at 50 mg L^−1^ Ag-NPs for the cultivar ‘Canino’ and 75 mg L^−1^ for the cultivar ‘Mirlo Rojo’, where the maximum accumulation of silver ions and nutrients in plant tissues was also recorded [86]. However, the application of the same concentration of Ag-NPs 25–45 nm in size during the proliferation of chrysanthemum (*Chrysanthemum* × *grandiflorum* (Ramat.) Kitam.) cultivars resulted in a 2.5–6.0-fold decrease in the MF of leaf explants, depending on the cultivar, while increasing plant polymorphism up to 32% [87]. For nodal segments of stevia, Ag-NPs 25–45 nm in size increased the MF by 41% and also improved growth parameters and total chlorophyll content [56]. For successful proliferation of *Maerua oblongifolia* Forssk. (A. Rich.), Ag-NPs 6–24 nm in size, obtained by green synthesis and applied at a concentration of 20 mg L^−1^, were used, resulting in a 43% increase in MF and higher chlorophyll and antioxidant enzyme levels [58]. Larger Ag-NPs (80–100 nm), chemically synthesized and applied at a concentration of 12 mg L^−1^, did not cause a significant increase in MF in *Musa acuminata* L. cultures, but stimulated increases in chlorophyll and proline content [88]. In lavender (*Lavandula angustifolia* Mill.) microshoot culture, Ag-NPs 22–26 nm in size at concentrations of 2–5 mg L^−1^ also stimulated antioxidant enzyme activity [78,79], whereas at 10 mg L^−1^ they contributed to a 9.5% increase in essential oil content [73]. In the same experiment, Au-NPs 22–32 nm in size at 10 mg L^−1^ increased antioxidant enzyme activity, whereas at 50 mg L^−1^ they reduced essential oil content by 17%; however, the ratio of its components shifted toward higher levels of 1,8-cineole and borneol, key precursors of camphor [78,79].

Au-NPs also substantially affect micropropagation efficiency. During clonal propagation of giant bamboo (*Dendrocalamus asper* (Schult. & Schult. f.) Backer ex K. Heyne), it was shown that the surface coating, which determines nanoparticle charge, as well as Au-NP concentration, differentially affected proliferation [89]. At a concentration of 154 mg L^−1^, citrate-coated Au-NPs with a negative charge increased the shoot proliferation coefficient by 57%, enhanced chlorophyll content, and increased antioxidant activity, outperforming CTAB-coated Au-NPs with a positive surface charge. Transcriptomic analysis revealed enrichment of differentially expressed genes in pathways associated with cell division and hormonal, particularly cytokinin, signaling, providing a molecular explanation for the stimulatory effects observed at optimal concentrations [89]. In a similar study, comparable results were obtained during shoot proliferation of *Nardostachys jatamansi* (D. Don) DC., although at substantially lower NP concentrations of 22–23 mg L^−1^ [90].

Selenium nanoparticles (Se-NPs) 50–100 nm in size were applied in the tissue culture of *Sarcocornia fruticosa* (L.) A.J. Scott to stimulate secondary metabolism and stabilize culture growth under saline conditions (700 and 1000 mM NaCl) [88]. Supplementation of the medium with 100 mg L^−1^ Se-NPs improved plant growth, increased K^+^, Na^+^, and Mg^2+^ contents, enhanced the K^+^/Na^+^ ratio, reduced lipid peroxidation, and improved stress tolerance. The use of Se-NPs as elicitors stimulated the production of phenolic compounds and isorhamnetin.

During in vitro cultivation of vanilla (*Vanilla planifolia* Andrews) in RITA bioreactors on liquid MS medium supplemented with SiO_2_-NPs at concentrations of 0, 50, 100, and 150 mg L^−1^, 150 mg L^−1^ was identified as the optimal concentration among the tested treatments. At this concentration, the MF increased from 2.8 to 5.1 shoots per explant, while shoot length and leaf number also increased significantly [91]. Notably, SiO_2_-NP application stimulated root initiation, resulting in the formation of up to five roots per plant by the end of the multiplication stage. This allowed the separate rooting stage to be omitted and substantially shortened the cultivation period, with a survival rate of 98%. Thus, the use of SiO_2_-NPs enabled the development of a more efficient protocol for vanilla propagation. However, it should be noted that 150 mg L^−1^ was the highest concentration tested in this study; therefore, the true optimal dose of SiO_2_-NPs for *V. planifolia* remains undetermined.

**Table 2:** Application of NPs at the in vitro multiplication stage.

NPs	Size, nm	Synthesis Method	Plant	Explant	Concentration, mg∙L^−1^	Experimental Conditions	Experiment Results	Reported Adverse Effects/Additional Information	Ref.
ZnO	25	Physical synthesis (microwave-assisted solvothermal synthesis)	*Chrysanthemum × morifolium* (Ramat.) Hemsl.	Internodes from previously cloned seedlings	500	MS (2× Fe, Ca) + 0.6 mg L^−1^ BA + 2 mg L^−1^ IAA	↑ shoot regeneration efficiency (+92% for ‘UTP Burgundy Gold’ and +7.4% for ‘UTP Pinky Gold’), ↑ pigment concentration for ‘UTP Burgundy Gold’ (tens of times), ↓ anthocyanin concentration (2.5 times) for ‘UTP Pinky Gold’	↓ anthocyanin concentration in ‘UTP Pinky Gold’; response was cultivar-dependent	[20]
ZnO	10–50	Green synthesis using extract of *Ochradenus arabicus* S. Chaudhary, D. Hillcoat & A.G. Miller	*Maerua oblongifolia* Forssk. (A.Rich.)	Micropropagated shoots	5	MS	↑ number of shoots (2.3 times), ↑ shoot length (3.2 times), ↑ FW (2.6 times), ↑ DW/FW ratio (+14%), ↑ chlorophyll concentration *a* and *b* (3 times), ↑ carotenoids (2.2 times), ↑ total protein (b 5 paɜ), ↑ SOD (11 times), ↑ CAT (4.5 times), ↑ GR (2.3 times), ↓ MDA (−28%)	↓ MDA; no adverse effect was reported at the optimal concentration	[81]
ZnO	26–98	Green synthesis using extract of *Convolvulus arvensis* L.	*Reseda lutea* L.	Shoots	15	MS + 0.4 mg L^−1^ BA	↑ FW (+35.4%), ↑ shoot length (+80%), ↑ number of shoots (+75%), ↑ chlorophyll concentration (+36.4%), ↑ total protein (+10.7%), ↑ sugar (+78.6%), ↑ TFD (+6.9%)	30 mg L^−1^: ↑ SOD and ↑ proline, indicating moderate stress. 60 mg L^−1^: ↓ shoot biomass (−29.39%); ↓ total chlorophyll (−40.78%); ↓ total protein; ↓ TPC and TFC; ↑ TBARS and ↑ proline; ↑ GR, SOD, and APX, indicating oxidative stress. Genetic stability of callus and shoots was confirmed by flow cytometry	[82]
					30		↑ FW (+17.4%), ↑ shoot length (+43%), ↑ number of shoots (+50%), ↑ chlorophyll concentration (+13.6%), ↑ total protein (+26.2%), ↑ sugar (+70.6%), ↑ TPC (+4.6%), ↑ TFD (+12.8%), ↑ SOD (b 2 paɜa), ↑ proline (+59.5%)		
CuO	≤50	Chemical synthesis (Sigma-Aldrich)	*Ajuga multiflora* Bunge	Leaves	5	MS + 2 mg L^−1^ BA + 0.5 mg L^−1^ NAA	↑ number of shoots (+24%), ↑ phytosterol (+20%), ↑ clerosterol (+22%).	10 mg L^−1^: ↓ number of shoots (−43%). Higher concentrations progressively reduced MF; >20 mg L^−1^ induced oxidative stress and phytotoxicity	[84]
					10		↑ FW (+10%) ↓ number of shoots (−43%), ↑ sterols (+5–7%)		
Ag	50–60	Chemical synthesis	*Origanum petraeum* Danin	Micropropagated shoots	50	MS + 0.5 mg L^−1^ GA3	↑ number of shoots (2.4 times—6.8 per explant), ↑ shoot length (+13.3%), ↑ number of roots (+46%), ↑ callus diameter (+79%) ↑ sesquiterpene (Caryophyllene Oxide (28.5%) and β-Caryophyllene (21.9%)), ↑ α-Humulene (14.7%) and Humulene Epoxide II (11.4%), ↑ minor constituents such as Geranial (2.9%) and Italicene Epoxide (2.9%).	TEM confirmed Ag-NP uptake/translocation in tissues; the optimal growth response was observed at 100 mg L^−1^, whereas 50 mg L^−1^ was more relevant for sesquiterpene accumulation	[85]
					100		↑ number of shoots (4 times—11.6 per explant), ↑ shoot length (+76%), ↑ number of roots (3.2 times), ↑ callus diameter (2.1 times)		
Cu	40–60	Chemical synthesis			50		↑ number of shoots (2.4 times—6.8 per explant), ↑ shoot length (+33%), ↑ number of roots (4.3 times), ↑ callus diameter (+79%), ↑ geranyl derivative biosynthesis: α-terpinyl acetate (29.2%), geranyl acetate (12.8%), geraniol (7.2%), β-caryophyllene (9.1%), γ-muurolene (7.2%), ↑ minor components: cyclopentadecanolide (3.8%) and cedroxyde (1.7%)	TEM confirmed the presence of Cu-NP aggregates in leaves; 50 mg L^−1^ was more effective than 100 mg L^−1^ for shoot number and metabolite modulation	
					100		↑ number of shoots (+14.3%—3.2 per explant), ↑ shoot height (+76%), ↑ number of roots (+87.5%), ↑ callus diameter (2.4 times)		
Ag	60–80	Chemical synthesis	*Prunus armeniaca* L. ‘Canino’	Micropropagated shoots	50	TIS QL macronutrients + DKW micronutrients and vitamins + 1.12 µM BA + 0.05 µM IBA	↑ MF (2.3 times), ↑ FW (3.3 times), ↑ DW (2.1 times), ↑ leaf surface area (2 times), ↑ concentration of nutrients	No negative effects were indicated for the optimal TIS treatment. The maximum metallic Ag content in shoots was 35.7 mg kg^−1^ at 50 mg L^−1^ Ag-NPs; in semi-solid medium, the maximum value was much lower, 5.0 mg kg^−1^ at 75 mg L^−1^. In semi-solid medium, Ag-NPs inhibited shoot growth and biomass accumulation	[86]
			*Prunus armeniaca* L. ‘Mirlo Rojo’		75		↑ MF (2 times), ↑ FW (2.4 times), ↑ DW (1.9 times), ↑ leaf surface area (3.2 times), ↑ concentration of nutrients		
Ag	23	Seeded-mediated growth method	*Chrysanthemum × grandiflorum* (Ramat.) Kitam. ‘Lilac Wonder’, ‘Richm + ond’	Leaves of cloned seedlings	50	MS + 2 mg L^−1^ BA + 2 mg L^−1^ IAA	↑ number of polymorphic plants (up to 28.2% and 32% depending on the variety)	↓ multiplication factor; ↑ proportion of polymorphic plants up to 28.2–32%, depending on cultivar, indicating reduced genetic stability of regenerants	[87]
Ag	25–45	Chemical method for obtaining concentrates of nanodispersions of zero-valent metals (Agrovit)	*Stevia rebaudiana* Bertoni	Shoot cuttings with 1 node	50	MS	↑ number of shoots (+41%), ↑ shoot length (+ 43%), ↑ DW (+23.5%), ↑ N content (+31%), ↑ Mg content (+28%), ↑ total chlorophyll (+37%), ↓ ability to form adventitious shoots (2.5–6 times), ↓ Fe content (−42%).	200 mg L^−1^: ↓ number and length of shoots. Ag content in tissues increased: 0.13 µg g^−1^ DW in the control; 95.23 µg g^−1^ DW at 100 mg L^−1^; 188.16 µg g^−1^ DW at 200 mg L^−1^. Ag-NPs were detected in stem epidermal cells, vascular bundles, intercellular spaces, leaf veins, and stomata	[56]
Ag	6–24	Green synthesis using extract of *Ochradenus arabicus chaudhary*, Hillc. & A.G.Mill.	*Maerua oblongifolia* Forssk. (A.Rich.)	Micropropagated shoots	20	MS	↑ number of shoots (+48%) ↑ shoot length (+67%), ↑ FW (+69%), ↑ DW/FW ratio (+6%), ↑ chlorophyll *a* (+84%) and *b* (+79%), ↑ total protein (+8%), ↑ proline (4 times), ↑ SOD (+17%), ↑ CAT (6 times)	No adverse effect was reported at 20 mg L^−1^	[58]
Ag	80–100	Chemical synthesis	*Musa acuminata* L.	Micropropagated shoots	12	MS + 12 μM BA + 40 mg L^−1^ cysteine HCl + 2 g L^−1^ gelrite	↓ microbial contamination, ↑ growing parameters, ↑ chlorophyll concentration (+25%), ↑ proline in shoots (+120%), ↑ lipid peroxidation level; however, limited effect on membrane stability index was observed	↑ lipid peroxidation, but with a limited effect on the membrane stability index	[88]
Ag	22–26	Chemical synthesis with two-stage microwave-convective heating	*Lavandula angustifolia* Mill.	Micropropagated shoots	2	MS + 0.2 mg L^−1^ IAA + 2 mg L^−1^ Kn	↑ APX (3.3 times), ↑ SOD (+20%), ↓ POX (2.5 times), ↓ polyphenols (−32%)	2 mg L^−1^: ↓ POX and ↓ polyphenols; 5 mg L^−1^: ↓ POX. Effects were strongly concentration-dependent	[78,79]
					5		↑ antioxidant activity: APX (4.5 times) and SOD (+45%), ↑ polyphenols (+51%), ↓ POX (14 times)		
					10		↑ essential oil content (+9.5%)		
Au	22–32				10		↑ antioxidant enzyme activity APX (+38%), ↑ polyphenols (+22%)	50 mg L^−1^: ↓ essential oil content (−17%); effects were concentration-dependent	[78,79]
					50		↑ borneol concentration (+16.5%), ↑ 1,8-cineole (+4.6%), ↓ essential oil content (−17%)		
Au	10–12	Chemical synthesis of Citrate-coated NPs	*Dendrocalamus asper* (Schult. and Schult. F.) Backer ex K. Heyne	Micropropagated shoots	154.4	MS +0.6 mg L^−1^ BA	↑ number of shoots (+57%), ↑ number of leaves (+44%), ↑ plant height (+71%), ↑ FW (+56%), ↑ DW (+58%), ↑ photosynthetic pigment concentration, ↑ antioxidant activity, ↑ TPC (+28%), ↓ ROS	Transcriptomic analysis indicated changes in pathways related to cell division and cytokinin signaling	[89]
			*Nardostachys jatamansi* (D.Don) DC.	Micropropagated shoots	23.2	1/2 MS + 1 mg L^−1^ IAA	↑ shoot growth (+22%), ↑ FW (+56%), ↑ number of roots (+56%), ↑ root length (+53%), ↑ photosynthetic pigment concentration—chlorophyll a and b, carotenoids (more than 2 times), ↑ inner concentration of hormones (GA3, IAA and ABA), ↑ antioxidant activity (TFD, TPC, DPPH and SOD)	No explicit negative effects were indicated for the optimal concentration; at non-optimal concentrations, CTAB-Au-NPs produced weaker morphogenic and biochemical responses than the optimal treatment	[90]
	16–22	Chemical synthesis of CTAB-coated NPs	*Dendrocalamus asper* (Schult. and Schult. F.) Backer ex K. Heyne	Micropropagated shoots	112.3	MS + 0.6 mg L^−1^ BA	↑ plant height (+66%), ↑ DW (+31%) ↑ antioxidant activity and synthesis of phenolic derivatives (+28%), ↑ photosynthetic pigment concentration	Citrate-stabilized Au-NPs induced stronger overall proliferative responses than CTAB-stabilized Au-NPs	[89]
			*Nardostachys jatamansi* (D.Don) DC.	Micropropagated shoots	22.5	1/2 MS + 1 mg L^−1^ IAA	↑ shoot height (+41%), ↑ FW (+56%), ↑ number of roots (+59%), ↑ root length (+ 49%), ↑ photosynthetic pigment concentration—chlorophyll a and b, carotenoids (more than 2 times), ↑ inner concentration of hormones (GA_3_, IAA and ABA), ↑ antioxidant activity (TFD, TPC, DPPH and SOD)	No explicit negative effects were indicated for the optimal concentration; at non-optimal concentrations, CTAB-Au-NPs produced weaker morphogenic and biochemical responses than the optimal treatment	[90]
Se	50–100	Not mentioned	*Sarcocornia fruticosa* (L.) A.J.Scott	segments of lateral shoots	100	Double strength MS containing 700 mM NaCl	↑ Na (2 times), ↑ K (+80%), ↑ K/Na ratio (2.5 times), ↑ Mg (2 times), ↑ GPX (2 times), ↑ PPO (2 times), compared to the positive control (700 mM NaCl salinization)	Under 1000 mM NaCl, stress persisted: ↓ FW, ↓ water content, ↓ photosynthetic pigments, and disrupted ion balance. Under 1000 mM NaCl + Se-NPs: ↑ Na^+^ relative to the saline control and ↓ PPO compared with the corresponding stress treatment. EDX confirmed the presence of Se-NPs in/on shoot tissues after 14 days of treatment	[91]
						Double strength MS containing 1000 mM NaCl	↑ FW (+10%) ↑ Na (2 times) ↑ K (2.5 times) ↑ K/Na ratio (+30%) ↑ Mg (+50%), ↑ isorhamnetin (4 times) compared to the positive control (1000 mM NaCl salinization)		
SiO_2_	≤50	Chemical synthesis (Sigma-Aldrich)	*Vanilla planifolia* Andrews	segments 1 cm long with 1 axillary bud	150	Liquid MS in RITA bioreactors	↑ number of shoots (+81%) ↑ shoot length (3.4 times) ↑ number of leaves (2 times) ↑ root initiation ↓ cultivation period (rooting stage is not required)	Rooting during the multiplication stage allowed omission of a separate rooting stage; acclimatization survival reached 98%	[92]

The table includes only adverse effects, control treatments, genetic stability data, and other safety-related observations that were explicitly reported in the original sources. If ionic controls, residual amounts of NPs/metals, genetic stability, abnormal morphology, acclimatization survival, or long-term growth parameters are not indicated for a particular study, this means that these parameters were not reported in the corresponding source. Abbreviations: ↑, increase/stimulation; ↓, decrease/inhibition; MS—Murashige and Skoog medium; QL—Quoirin and Lepoivre medium; BA—6-benzyladenine; TDZ—thidiazuron; GO—graphene oxide; MWCNTs—multi-walled carbon nanotubes; CAT—catalase; POD—peroxidase; PAL—phenylalanine ammonia-lyase; APX—ascorbate peroxidase; TPC—total phenolic content; IAA—indole-3-acetic acid; NAA—1-Naphthaleneacetic acid; IBA—Indole-3-butyric acid.

Thus, at the shoot multiplication stage, NP effectiveness should be evaluated not by a single universal indicator but by a set of stage-specific parameters, including the MF, number and length of shoots, morphological quality of regenerants, frequency of abnormal development, signs of vitrification, rooting capacity, and subsequent ability to acclimatize. Increased chlorophyll content, antioxidant enzyme activity, or proline levels may accompany improved physiological status of plants, but these parameters may also reflect a stress response to NP exposure. Therefore, such responses should be interpreted separately from a direct improvement in micropropagation efficiency. This is particularly important for Ag-NPs, which in some cases stimulate shoot proliferation and growth but in others may inhibit growth, increase the proportion of polymorphic plants, or lead to silver accumulation in tissues. Consequently, the application of NPs at the multiplication stage requires not only dose optimization but also assessment of regenerant quality, genetic stability, and residual content of the nanomaterial or corresponding metal.

### 2.3. Application of NPs in In Vitro Rooting Stage

To stimulate root formation after the in vitro multiplication stage, microcuttings are transferred to a culture medium with a modified basal composition, characterized by reduced salt and carbohydrate content and supplemented with auxins, such as indole-3-acetic acid, naphthaleneacetic acid, or indole-3-butyric acid, at low concentrations of 1–5 mg L^−1^ [93]. Cultivation is carried out for 3–4 weeks. It has been established that the induction of morphogenetic pathways in vitro is determined by the balance between endogenous auxin levels within the culture and exogenous auxin concentrations in the culture medium [94]. Moreover, the optimal auxin type and concentration for effective rooting are species-specific [94].

A limited number of studies have shown that NPs also have the ability to stimulate root formation (Table 3). For example, the effectiveness of CuO-NPs and ZnO-NPs in inducing root formation in stevia has been reported [60]: more than 90% of micro cuttings formed roots when treated with NPs at concentrations of 20 and 2 mg L^−1^, respectively. For root initiation in *Ajuga multiflora* Bunge, CuO-NPs at concentrations of 5–10 mg L^−1^ were the most effective, whereas higher concentrations of 20 mg L^−1^ and above inhibited root formation [84].

The effectiveness of Ag-NPs in stimulating both root formation and root elongation has also been demonstrated for *M. acuminata* [88].

### 2.4. Prospects for the Application of Nanoparticles in Plant Adaptation to Non-Sterile Conditions

Currently, information on the use of NPs during the rooting stage and subsequent adaptation to non-sterile conditions remains relatively limited. NPs with stress-protective properties appear to be the most promising for improving plant acclimatization. Such NPs should contribute to the stabilization of photosynthetic processes and stomatal function, activate mechanisms involved in oxidative stress responses, and strengthen the plant immune system. These include Se-NPs, Si-NPs, Mn-NPs, CH-NPs, CeO_2_-NPs, CuO-NPs, TiO_2_-NPs, Fe_2_O_3_-NPs, FeS-NPs, and others, whose effectiveness has been demonstrated in a number of experimental studies [34,35,36,37,38].

**Table 3:** Application of NPs for in vitro rooting.

NPs	Size, nm	Obtaining Method	Plant	Explant	Optimal Concentration, mg∙L^−1^	Experimental Conditions	Experiment Results	Reported Adverse Effects/Additional Information	Ref.
CuO	25–30	Chemical synthesis (coprecipitation method)	*Stevia rebaudiana* Bertoni	Micropropagated shoots from seeds	20	MS	The percentage of induced root formation −94%, ↑ SGS concentration (rebaudioside A (+25%), stevioside (+17%)), ↑ shoot length (+25%), ↑ number of roots (+29%), ↑ root length (+32%) ↑ number of nodes (+28%), ↑ number of leaves (+43%), ↑ FW (+12%), ↑ TPC (+56%), ↑ antioxidant activity (+20%), ↑ total reducing power (+20%) ↑ DPPH (+20%)	Phytotoxicity increased at concentrations above the optimum. 200 mg L^−1^: ↓ rooting to 60.8%; ↓ root length to 4.41 cm; ↓ number of roots to 6.02; ↓ leaf FW to 0.13 g. 2000 mg L^−1^: maximum phytotoxicity; ↓ rooting to 48.5%; roots were not formed; ↓ regenerant length to 10.4 cm; ↓ leaf FW to 0.08 g; ↓ rebaudioside A to 1.77% and ↓ stevioside to 0.25%; minimum antioxidant activity values	[60]
CuO	<50	Chemical synthesis (Sigma-Aldrich)	*Ajuga multiflora* Bunge	Tips of axillary shoots	5	MS + 2 BA + 0.5 NAA	↑ shoot induction (+24%)	20 mg L^−1^: ↓ number of roots; ↓ FW of regenerated plants. 40 mg L^−1^: complete inhibition of rooting; ↓ growth; leaf yellowing; pronounced phytotoxicity	[84]
					10		↑FW (+10%), ↓ number of shoots (−43%)		
ZnO	20–30	Chemical synthesis (coprecipitation method)	*Stevia rebaudiana* Bertoni	Micropropagated shoots from seeds	2	MS	↑ rooting induction (91%), ↑ SGS concentration (rebaudioside A (+25%), stevioside (+45%), ↑ shoot length (+43%), ↑ number of roots (+33%), ↑ root length (+43%), ↑ number of nodes (+33%), ↑ number of leaves (+26%), ↑ FW (+26%), ↑ TPC (+58%), ↑ TFD (+50%), ↑ total reducing power (+14%), ↑ DPPH (+10%)	200–2000 mg L^−1^: ↓ biomass; ↓ antioxidant activity; ↓ steviol glycoside accumulation. 2000 mg L^−1^: maximum phytotoxicity; ↓ rebaudioside A to 1.22%; ↓ stevioside to 0.21%	[60]
Ag	80–100	Chemical synthesis	*Musa acuminata* L.	Micropropagated shoots	12	MS + 5 μM 2iP + 0.1 μM IBA	↑ root length ↑ number of roots (3 times)	↑ lipid peroxidation; no pronounced decrease in the membrane stability index at 12 mg L^−1^	[88]

The table includes only adverse effects, control treatments, genetic stability data, and other safety-related observations that were explicitly reported in the original sources. If ionic controls, residual amounts of NPs/metals, genetic stability, abnormal morphology, acclimatization survival, or long-term growth parameters are not indicated for a particular study, this means that these parameters were not reported in the corresponding source. Abbreviations: ↑, increase/stimulation; ↓, decrease/inhibition; MS—Murashige and Skoog medium; BA—6-benzyladenine; TDZ—thidiazuron; GO—graphene oxide; MWCNTs—multi-walled carbon nanotubes; CAT—catalase; POD—peroxidase; PAL—phenylalanine ammonia lyase; APX—ascorbate peroxidase; TPC—total phenolic content.

## 3. Application of Nanoparticles to Stimulate the Production of Plant Secondary Metabolites

One of the most important areas of plant biotechnology is the production of plant-derived secondary metabolites, whose structural complexity often limits the possibilities of their chemical synthesis [95]. The main objective of this field is to increase the content of valuable biologically active compounds in plant material. Compounds such as phenolics, pigments, alkaloids, steroids, glycosides, essential oils, and other primary and secondary metabolites of plant origin serve as raw materials for the production of pharmaceuticals, medicinal products, and cosmetics [15,16]. Four major groups of secondary metabolites are distinguished: nitrogen-containing compounds, including alkaloids and glycosides; aromatic compounds with hydroxyl groups, including phenols, flavonoids, coumarins, stilbenes, lignins, and tannins; sulfur-containing compounds, including glucosinolates, phytoalexins, and glutathione; and terpenes, including monoterpenes, sesquiterpenes, and diterpenes [17].

The commercial application of plant tissue culture for the production of valuable biologically active compounds has not yet become widespread worldwide; however, it already plays a significant role in the pharmaceutical industry. Current studies show that in vitro culture systems, such as cell suspension cultures, callus cultures, and hairy root cultures, have considerable potential for the production of secondary metabolites for various applications. Cell suspension culture represents a more industrially oriented, although costly, approach to the cultivation of individual plant cells in liquid medium and requires strictly controlled conditions provided by bioreactors [96,97].

The choice of cultivation method depends on the plant species [15], since the synthesis of target compounds may not occur throughout the entire plant but may instead be localized in specific organs, such as roots, leaves, or fruits [98].

It has been established that the production of plant secondary metabolites requires stress and defense-related signals [18,99], which enables various NPs to act as inducers of their biosynthesis [23]. In addition, NPs may serve as precise regulators of metabolic processes and the biosynthesis of target compounds [55].

### 3.1. Application of Nanoparticles in Callus Culture

The use of callus cells, i.e., undifferentiated totipotent cells, cultivated on various nutrient media supplemented with growth regulators and/or elicitors, significantly reduces the required biomass and accelerates the production of target metabolites. It was established that the phytochemical profiles of leaf, stem, and callus extracts of *Syringa vulgaris* L. were highly similar [100], indicating that the use of callus tissue does not compromise the quality of the target compounds. Moreover, sterile cell cultures obtained in vitro share a common genetic background.

In callus culture, NPs can function as elicitors: at low concentrations, they stimulate cell growth and enhance the production of secondary metabolites, whereas at higher concentrations they may inhibit growth or even induce plant cell death [24].

To stimulate callus formation, plant growth regulators such as 2,4-D, BA, and Kn are usually added to the basal nutrient medium at species-specific concentrations. The application of NPs synthesized by different methods and characterized by different sizes and concentrations offers several advantages, including economic ones, since the cost of NPs may in some cases be lower than that of hormonal preparations. Typical responses of callus cultures to NP supplementation (Table 4) include increased FW and DW, as well as elevated levels of phenolic compounds and other secondary metabolites.

In addition to stimulating metabolite synthesis, NPs exert other beneficial effects in callus cultures, including mitigation of salt stress [101], stimulation of cell growth and division [82,102,103,104,105], and enhancement of antioxidant status [22,27,28,31,101,102,105,106,107,108]. Regardless of the synthesis method, most studies report that the addition of NPs to the nutrient medium promotes the accumulation of phenolic compounds in callus cells [82,108,109,110,111,112].

For example, in large-scale production of phenolic acids and flavonoids using *Perilla frutescens* L., Cu- and Ag-NPs were recommended as effective elicitors of secondary metabolite biosynthesis [107]. Treatment with Cu-NPs (15–60 nm) at a concentration of 100 mg L^−1^ resulted in the highest levels of ferulic and rosmarinic acids and phenylalanine ammonia-lyase activity. Application of Ag-NPs (20–40 nm) at 100 mg L^−1^ provided maximum accumulation of caffeic acid and rutin and also enhanced antioxidant activity.

In callus cultures of three *Vigna radiata* L. cultivars (Mung NCM-13, MgAT-7, and MgAT-4), the addition of biosynthesized Cu-NPs (11.5 nm) or ZnO-NPs (37.8 nm) at 0.5 mg L^−1^ to the nutrient medium stimulated the production of phenolic compounds and glycosides [28]. Both types of NPs used as nanoelicitors significantly increased total phenolic content by 23–26% in all cultivars and glycoside content by 25–50%, depending on genotype.

In callus cultures of *Bacopa monnieri* (L.) Pennell treated with chemically synthesized ZnO-NPs (18–42 nm) at concentrations of 0, 0.25, 0.50, 0.75, and 1 mg L^−1^, a linear relationship was observed between bacoside A yield and NP concentration [113]. Treatment with 1 mg L^−1^ resulted in an approximately twofold increase in bacoside A content and was accompanied by reduced expression of the HMG-CoA reductase gene, indicating the possible involvement of ZnO-NPs in the isoprenoid biosynthetic pathway.

The biological effects of NPs may depend not only on the synthesis method but also on the source material used. In callus cultures derived from *Silibium marianum* (L.) Gaertn. seeds, ZnO-NPs obtained by green synthesis (using extracts from both callus (C-ZNPs) and green parts (G-ZNPs) of the plant) [27]. C-ZNPs showed better results for seed germination (65%), fresh weight (FW), dry weight (DW), and root and shoot length. C-ZNPs and G-ZNPs) were evaluated [27]. G-ZNPs provided more pronounced accumulation of phenolic and flavonoid compounds and proteins, accompanied by maximum antioxidant activity (99%). Smaller ZnO-NPs (15–25 nm), synthesized using an aqueous leaf extract of *Ricinus communis* L., were applied at concentrations of 10, 20, 30, 40, and 50 mg L^−1^ in callus cultures derived from seedlings of *Delonix elata* (L.) Gamble [103]. These treatments stimulated secondary metabolite production, with 30 mg L^−1^ identified as the optimal concentration for increasing callus FW, total phenolic and flavonoid contents, and the levels of gallic acid, quercetin, hesperidin, and rutin. The predominant compound detected was bis(2-ethylhexyl) phthalate.

In callus cell cultures of *Linum usitatissimum* L., biosynthesized ZnO-NPs (35 nm) were applied at the optimal concentration of 100 mg L^−1^ and introduced into the nutrient medium on day 0, on days 0 and 15, or on days 0 and 25 of cultivation [108]. Repeated stimulation of cell cultures on days 0 and 15 resulted in the highest FW accumulation and lignan production, whereas stimulation on days 0 and 25 provided the greatest DW, as well as enhanced synthesis of phenolic compounds, flavonoids, and neolignans. ZnO-NPs (26–98 nm), synthesized by a green method using *Convolvulus arvensis* L. extract, increased the FW of *Reseda lutea* L. callus by 71, 63, and 25% at concentrations of 15, 30, and 60 mg L^−1^, respectively [82]. The maximum increase in total phenolic and flavonoid contents in callus (15.5%) was achieved at 60 mg L^−1^. Oxidative stress was observed already at 30 mg L^−1^, as indicated by increased SOD activity and proline content, whereas 60 mg L^−1^ exerted a toxic effect, as confirmed by increased ascorbate peroxidase (APX) activity and thiobarbituric acid reactive substances (TBARS) levels. Flow cytometry analysis of callus confirmed its genetic stability under all treatment conditions.

In addition to zinc oxide, graphene oxide-coated zinc sulfide NPs (GO–ZnS) were also evaluated in callus cultures at concentrations of 100, 300, and 450 mg L^−1^ for the elicitation of *Thymus daenensis* Celak under salt stress (150 mM NaCl) and non-stress conditions [101]. All GO–ZnS treatments increased H_2_O_2_ levels and enhanced antioxidant activity. Under salt stress, 100 mg L^−1^ GO–ZnS-NPs provided the highest total phenolic content (TPC), increased phenylalanine ammonia-lyase (PAL), APX, and catalase (CAT) activities, and enhanced total antioxidant capacity. The greatest increase in total flavonoid content (TFC) and thymol accumulation was observed at 300 mg L^−1^.

The application of Ag-NPs has been studied most extensively in callus cultures, particularly as stimulators of plant secondary metabolite biosynthesis. Studies of different combinations of 40 nm Ag-NPs with plant growth regulators (2,4-D and BA) in the callus culture of *Caralluma tuberculata* N.E. Brown showed that NPs at different concentrations increased callus biomass by cell division [114]. The most effective treatment for biomass accumulation was 60 µg L^−1^ Ag-NPs in combination with 0.5 mg L^−1^ 2,4-D and 3 mg L^−1^ BA, whereas 90 µg L^−1^ Ag-NPs were optimal for the accumulation of phenolic compounds, flavonoids, and antioxidant activity.

Ag-NPs biosynthesized using *Hibiscus tiliaceus* L. leaf extract and characterized by a smaller particle size of 21–26 nm stimulated callus growth in *Rumex nervosus* Vahl and exhibited antibacterial and antifungal activity at concentrations of 5, 10, 20, and 40 mg L^−1^ [115]. However, treatment with 40 mg L^−1^ Ag-NPs induced polymorphism in 12.5% of cases, although the culture remained relatively stable. Ag-NPs of similar size (25 nm), biosynthesized using *Parthenium hysterophorus* L. extract, were studied in rice (*Oryza sativa* cv. IR64), which is characterized by low in vitro regeneration capacity [106]. Ag-NPs at concentrations of 5 and 10 mg L^−1^ stimulated callus growth and rhizogenesis, with 5 mg L^−1^ being optimal for regeneration and improvement of antioxidant status.

**Table 4:** Application of NPs in callus culture.

NPs	Size, nm	Obtaining Method	Plant	Explant	Concentration mg∙L^−1^	Experimental Conditions	Experiment Results	Reported Adverse Effects/Additional Information	Ref.
Cu	15–60	Chemical synthesis	*Perilla frutescens* (L.)	Callus obtained from a nodal explant	50	MS + 1 mg L^−1^ 2,4-D + 1 mg L^−1^ BA	↑ FW (+75%), ↑ DW (+13%), ↑ MDA (2.5 times), ↑ SOD (2 times).	50–100 mg L^−1^: ↑ MDA. 100 mg L^−1^: ↑ H_2_O_2_; ↓ FW; ↓ DW	[107]
					100		↑ TPC, total anthocyanins, ↑ SOD, MDA (2.5 times), ↑ H_2_O_2_ (+45%), ↑ ferulic acid (1.6 times), ↑ rosmarinic acid (2 times), ↑ Phenylalanine ammonia lyase (1.5 times ↓ FW (−14%), ↓ DW (−23%).		
CuO	11.5	Green synthesis using *Nigella sativa* L. extract	*Vigna radiata* L. (Mung NCM-13, MgAT-7 и MgAT-4)	Callus	0.5	MS + 3 mg L^−1^ 2,4-D	↑ TPC (+20–27% depending on the variety) ↑ glycoside content by 1.5 and 2% (for varieties MgAT-7 and MgAT-4, respectively)	0.5 mg L^−1^: explant browning. CuO-NPs did not increase glycoside content in Mung NCM-13; in MgAT-7 and MgAT-4, the increase in glycosides was weak (+1.5–2%). The response was genotype-dependent	[28]
ZnO	38				0.5		↑ TPC (+5, 20 and 16% for Mung NCM-13, MgAT-7 and MgAT-4) ↑ glycoside content (+48, 42 and 28% for Mung varieties NCM-13, MgAT-7 and MgAT-4)	>0.5 mg L^−1^: explant browning. The increase in glycosides was genotype-dependent: Mung NCM-13 > MgAT-7 > MgAT-4	
ZnO	18–42	Chemical precipitation method	*Bacopa monnieri* (L.) Pennell	Callus	1	MS + mg L^−1^ NAA	↓ transcription of the HMG-CoA reductase gene (−40%), ↑ bacoside A (2 times)	↓ transcription of the HMG-CoA reductase gene	[113]
ZnO	64–70	Green synthesis using *S. marianum* callus	*Silybum marianum* (L.) Gaertn.	Seeds, shoots	0.15	MS + 5 mg L^−1^ BA + 1 mg L^−1^ NAA	↑ TPC (+30%), ↑ protein (2.5 times)	No adverse effects were indicated for 0.15 mg L^−1^	[27]
	55–60	Green synthesis using *S. marianum* green parts					↑ protein (2 times)	No explicit adverse effects were indicated for 0.15 mg L^−1^	
ZnO	15–25	Green synthesis using *Ricinus communis* L.	*Delonix elata* (L.) Gamble	Seeds, callus	30	MS + 2.5 kmol L 2,4-D + 1 kmol L^−1^ BA	↑ callus FW (+24%), ↑ TPC (+46%), ↑ TFD (+39%), ↑ gallic acid (+49%), ↑ quercetin (+60%), ↑ hesperidin (2.8 times), ↑ rutin (2 times). The main compound is bis (2-ethylhexyl) phthalate (38.67%), and without addition—octadecanoic acid: 2-propenyl ether (10%), ethenyl ether (9.8%), bis (2-ethylhexyl) phthalate (9%)	The metabolite profile shifted; bis(2-ethylhexyl) phthalate became the predominant detected compound	[112]
ZnO	35	Green synthesis using *Linum usitatissimum* L.	*Linum usitatissimum* L.	Callus	100	MS + 1 mg L^−1^ NAA	↑ FW (10 times), ↑ lignan synthesis: ↑ secoisolariciresinol diglucoside (3.2 times), ↑ lariciresinol diglucoside (5.7 times);	No explicit toxic effects were indicated. Days 0 + 15: maximum FW and lignans. Days 0 + 25: lower FW/lignans, but maximum DW, TPC, TFD, and neolignans	[108]
						Double NP treatment: 0 and 15th day			
						Double NP treatment: 0 and 25th day	↑ DW (2.2 times), ↑ TPC (15 times), ↑ TFD (8.2 times), ↑ production of neolignans, ↑ dehydrodiconiferyl alcohol glucoside (11.7 times), ↑ guaiacylglycerol-β-coniferyl glucoside alcohol ester (21 times)		
ZnO	26–98	Green synthesis using *Convolvulus arvensis* L.	*Reseda lutea* L.	Callus	15	MS + 0.3 mg L^−1^ 2,4-D + 0.1 mg L^−1^ BA	↑ FW (+70%), ↑ total protein (+36.4%), ↑ sugar (+22.6%), ↑ TPC (+6.7%), ↑ TFD (+13.7%)	Oxidative-stress responses at 30 mg L^−1^; 60 mg L^−1^ showed toxic/stress-related effects, including ↑ TBARS and ↑ APX; callus genetic stability was confirmed by flow cytometry	[82]
					30		↑ FW (+62%), ↑ total protein (+14.9), ↑ sugar (+6.7%), ↑ TPC (+1.1%), ↑ TFD (+19.8%), ↑ SOD (+56%), ↑ proline (2.2 times)		
					60		↑ FW (+25%), ↑ sugar (+40%), ↑ TPC (+1.5%), ↑ TFD (+58%), ↑ TBARS (+37%), ↑ SOD (3 times), ↑ proline (2.8 times), ↑ APX (3.6 times)		
GO-ZnS	100	Chemical method (Hammers and Offman)	*Thymus daenensis* Celak	Leaf callus	100	1/2 MS 3 mg L^−1^ 2,4-D + 1.5 mg L^−1^ Kn	↑ TPC (+68%), ↑ TFD (+66.2%), ↑ PAL (+38.5%), ↑ APX (2 times), ↑ CAT (4.7 times), ↑ FRAP (+76.2%), ↑ H_2_O_2_ (+57.3%), ↑ DPPH (+19.6%), ↑ thymol (4.3 times)	↑ H_2_O_2_ under all GO–ZnS treatments	[101]
					300		↑ TPC (2.9 times), ↑ TFD (2 times), ↑ PAL (5 times), ↑ H_2_O_2_ (+92.5%), ↑ APX (3 times), ↑ DPPH (+35.3%), ↑ CAT (7 times), ↑ FRAP (6 times), ↑ thymol (17.5 times)		
					100	1/2 MS 3 mg L^−1^ 2,4-D + 1.5 mg L^−1^ Kn + 100 mM NaCl (salt stress)	↑ TPC (2.5 times), ↑ TFD (+25%), ↑ PAL (7 times), ↑ APX (2.5 times), ↑ CAT (+26.1%), ↑ FRAP (6.2 times), ↑ H_2_O_2_ (2.1 times), ↑ DPPH (+16.4%)		
					300		↑ TPC (+13.9%), ↑ TFD (+50%), ↑ H_2_O_2_ (2.5 times), ↑ APX (2.7 times), ↑ PAL (+27.5%), ↑ DPPH (+32.8%), ↑ CAT (−34.8), ↑ FRAP (2.1 times), ↑ thymol (2.5 times)		
Ag	20–40	Green synthesis using *P. frutescens* leaf extract	*Perilla frutescens* L.	Callus obtained from a nodal explant	50	MS + 1 mg L^−1^ 2,4-D + 1 mg L^−1^ BA	↑ FW (+7%), ↑ MDA (+15%), ↑ APX (+29%)	↑ MDA.	[107]
					100		↑ MDA (+27%), ↑ APX (+30%), ↑ caffeic acid (0.6 mg g^−1^ DW), ↑ routine (1.1 mg g^−1^ DW), ↑ DPPH (+10%), ↓ FW (−12%)	↓ FW	
Ag	40	Chemical synthesis	*Caralluma tuberculata* N.E. Brown	Callus	0.06	MS + 0.5 mg L^−1^ 2,4-D + 3 mg L^−1^ BA	↑ callus proliferation, ↑ FW (6 times), ↑ DW (3.7 times)	No explicit adverse effects were indicated at 0.06–0.09 mg L^−1^	[114]
					0.09		↑ TPC and TFD (1.5 times), ↑ PAL (1.7 times), ↑ antioxidant capacity (+20%); ↑ antioxidant enzyme activity: SOD (2.4 times), POD (1.7 times), CAT (2.3 times) and APX (1.9 times)		
Ag	21–26	Green synthesis using *Hibiscus tiliaceus* L. leaf extract	*Rumex nervosus* Vahl	Callus	40	MS	↓ radial fungi growth (−42.6%), ↑ bacteria inhibition (+98.1%), improving callus growth while maintaining its genetic stability	Polymorphism was 12.5%, indicating relatively high but incomplete genetic stability	[115]
Ag	25	Green synthesis using *Parthenium hysterophorus* L. extract	*Oryza sativa* cv. IR64	Embryogenic callus	5	MS + 2.5 mg L^−1^ 2,4-D	↑ callus regeneration (+20%), ↑ regeneration frequency (+50%), ↓ ethylene in plant tissues, ↓ MDA (−64%), ↓ proline (−10%) The length and number of roots did not change.	No explicit adverse effects were indicated at 5 mg L^−1^	[106]
Ag	30	Chemical reduction method (polyol method)	*Foeniculum vulgare* Mill.	Leaf fragments, Callus	20	MS + 2 mg L^−1^ 2,4-D	The callus is compact, yellow-brown, ↑ FW (7.3 times), ↑ fatty acid content (+2 orders of magnitude), ↑ Vitamin E (13 times), ↑ Vitamin D3 (17 times), ↑ Vitamin A (46 times), ↑ Vitamin K (18 times), ↑ Vitamin B1 (595 times), ↑ Vitamin B6 (4.3 times)	No explicit toxic effects were indicated at 20–40 mg L^−1^.	[104]
					40	MS	The callus is compact, greenish, ↑ FW (2.8 times), ↑ fatty acid content (+1 orders of magnitude), ↑ Vitamin E (9.7 times), ↑ Vitamin D3 (11.3 times), ↑ Vitamin A (31.9 times), ↑ Vitamin K (8.9 times), ↑ Vitamin B1 (548.5 times), ↑ Vitamin B6 (3.4 times)	↓ effectiveness compared with 20 mg L^−1^ for most fatty acids and vitamins;	
Ag	12–30	Green synthesis using *Eclipta alba* L.	*Eclipta alba* L.	Callus	5	MS + 1 mg L^−1^ BA	↑ FW (4.8 times), ↑ DW (6 times), ↑ TPC (7 times), ↑ TFD (3 times), ↑ DPPH (+8%), ↑ antioxidant capacity (7.7 times), ↑ total reducing power (4.9 times)	No explicit toxic effects were indicated	[105]
					10		↑ FW (2.8 times), ↑ DW (4.3 times), ↑ TPC (7 times), ↑ TFD (2 times), ↑ total protein (+80%), ↑ DPPH (+10%), ↑ antioxidant capacity (6.5 times), ↑ total reducing power (4.1 times)		
Ag-Cit-L-Cys	7–9	Chemical synthesis	*Populus nigra* L.	Callus	2.5	MS + 0.1 mg L^−1^ 2.4-D	↑ lipid peroxidation level (+30%), ↑ APX (6 times), ↑ CAT (2 times), ↓ FW (−39%), ↓ protein (−44%).	↑ lipid peroxidation; ↓ FW; ↓ protein; in the original study, nanoparticles induced stronger toxicity than AgNO_3_	[116]
AgAu (1:3)	25–35	Chemical synthesis	*Prunella vulgaris* L.	Callus	30	MS + 2 mg L^−1^ NAA	↑ total protein (2 times), ↑ antioxidant enzyme activity: ↑ SOD (2 times), ↑ TPC (+35%), ↑ TFD (+39%).	No explicit toxic effects were indicated	[117]
Fe_3_O_4_	10–20	Chemical synthesis (co-precipitation)	*Hypericum perforatum* L.	Leaf callus	4	MS + 0.5 mg L^−1^ 2,4-D, + 2 mg L^−1^ Kn	↑ plant height (+60%), ↑ root length (+114%), ↑ number of shoots (2.8 times), ↑ FW (3 times), ↑ regeneration efficiency (+51%), ↑ hypericin (+67%), ↑ TPC + TFD (+80%)	No explicit adverse effects were indicated at 4 mg L^−1^ Fe_3_O_4_-NPs	[103]
FeO_3_-CTs	50–100	Chemical synthesis (co-precipitation)	*Nigella sativa* L.	Callus	100	MS + 2 mg L^−1^ 2,4-D + 1 mg L^−1^ BA	↑ FW 6.6 g (+18%) under R and 5 g (+67%) under D, ↑ DW 1.3 g (+18%) under R and 1 g (+25%) under D, ↑ DPPH (+22%), ↑ quercetin (+18%), ↑ carbohydrates (+67%) under D, ↑ TPC (+65.3%) under R, ↑ TFD (+50%) under R, ↑ amino acids (+45.7%), ↑ thymoquinone (+80%) under R and B	No explicit toxic effects were indicated	[102]
					200	MS + 0.5 mg L^−1^ 2,4-D + 3 mg L^−1^ BA, 3 types of light were used: dark (D), white (W), blue (B) and red (R) with an intensity of 2000 lux	↑ FW 5.4 g (+80%) under D, ↑ DW 1.1 g (+26%) under D, ↑ DPPH (+24%), ↑ quercetin (+17%), ↑ carbohydrates (+33%) under D, ↑ TPC (+53.6) under R, ↑ amino acids (+25%), ↑ thymoquinone (+80%) under B		
MnO	10–40	Green synthesis using *Syzygium cumini* (L.) Skeels. leaf extract	*Moringa oleifera* Lam.	Seeds, callus	10	MS + 2 mg L^−1^ 2,4-D + 0.3 mg L^−1^ Kn	↑ callus initiation rate from 72 to 95% (callus was larger, compact and green), ↑ rate of callus initiation (4 days instead of 9), ↑ FW (+20%), ↑ DW/FW (+11%).	No explicit toxic effects were indicated	[118]
TiO_2_	24.5	Chemical synthesis (Degussa P-25 mixture of anatase and rutile crystallites)	*Hordeum vulgare* L.	Mature embryos	60	MS	↑ cell division, ↑ cell size and callus growth of explants under dark conditions, Bactericidal effect without negative changes in callus quality	No explicit toxic effects were indicated	[119]
TiO_2_/perlite	14–23	Green synthesis using *H. perforatum* extract	*Hypericum perforatum* L.	Callus	100	MS + 1 mg L^−1^ 2,4-D, + 1 mg L^−1^ BA	↑ FW (+108%), ↑ number of shoots (+35%), ↑ alkaloids (10.7 times), ↑ TPC (from 0 to 1.8 mg L^−1^)	No explicit toxic effects were indicated	[31]
perlite	15–25	Physical synthesis		Callus	50	MS + 1 mg L^−1^ 2,4-D, + 1 mg L^−1^ BA	↑ FW (+104%), ↑ number of shoots (+42%), ↑ alkaloids (12.7 times), ↑ TPC (from 0 to 22 mg L^−1^)	No explicit toxic effects were indicated at 100 mg L^−1^	[31]
					100		Compounds that were not present in the control appeared (% from Total identification): ↑ aliphatic hydrocarbons (70.5%), ↑ fatty acids (4.4%), ↑ sesquiterpenes (1.9%), ↑ diterpenes (7.2%). FW and the number of shoots did not change		
Se	40–100	Green synthesis using *Allium sativum* L.	*Caralluma tuberculata* NE Brown	Callus	0.1–0.2	MS + 0.5 mg L^−1^ 2,4-D + 3 mg L^−1^ BA	↑ FW (3 times in light/dark mode), ↑ TPC (+40–50%), ↑ TFD (3 times), ↑ POD (3–4 times), ↑ SOD (4.5–5 times), ↑ DPPH (+40–50%), ↑ concentration of secondary metabolites (coumarins, gallic acid, caffeic acid, ferulic acid, catechin, querctin and rutin)	No explicit toxic effects were indicated	[120]

The table includes only adverse effects, control treatments, genetic stability data, and other safety-related observations that were explicitly reported in the original sources. If ionic controls, residual amounts of NPs/metals, genetic stability, abnormal morphology, acclimatization survival, or long-term growth parameters are not indicated for a particular study, this means that these parameters were not reported in the corresponding source. Abbreviations: ↑—Increase in indicators, ↓—Decrease in indicators, FW-fresh weight, DW—Dry weight, MS—Murashige and Skoog medium, TFD—Total flavonoid, TPC—Total phenolics content, NAA—1-Naphthaleneacetic acid, DPPH—1,1-diphenyl-2-picrylhydrazil, FRAP—Ferric Reducing Antioxidant Power, IBA—Indole-3-butyric acid, 2iP—N6-(delta-2-isopentenyl)adenine, GSH—Glutathione, GR—Glutathione reductase, GPX—Glutathione peroxidase, APX—Ascorbate peroxidase, CAT—Catalase, POD—Peroxidase, SOD—Superoxide dismutase, PAL—phenylalanine ammonia-lyase, MDA—Malondialdehyde, POX—peroxidase, ROS—reactive oxygen species, GA3—Gibberellic acid, IAA—indole-3-acetic acid, PPO—Polyphenol oxidase, TBARS—Thiobarbituric acid reactive substances, ABA—abscisic acid.

For callus culture of *Foeniculum vulgare* Mill., replacement of 2 mg L^−1^ 2,4-dichlorophenoxyacetic acid (2,4-D) with 40 mg L^−1^ Ag-NPs (30 nm) synthesized by the polyol method [117] resulted in a 2.8-fold increase in callus FW and a substantial increase in the levels of vitamins E, D_3_, A, K, B_1_, and B_6_ and fatty acids by an order of magnitude [104]. However, the combined application of 2,4-D and 20 mg L^−1^ Ag-NPs was even more effective, especially with respect to fatty acid content, which increased by two orders of magnitude.

The effects of Ag-NPs 12–30 nm in size at different concentrations (2, 4, 6, 8, 10, and 12 mg L^−1^), in combination with 1 mg L^−1^ BA and additional supplements, including ascorbic acid, adenine sulfate, arginine, and citric acid, on callus growth and secondary metabolite synthesis in *Eclipta alba* L. were also investigated [115]. The optimal concentration of 8 mg L^−1^ significantly increased callus FW and DW, as well as the production of phenolic compounds, flavonoids, total protein, antioxidant activity, and total reducing capacity. At 10 mg L^−1^, antioxidant activity reached its maximum value of 97%.

Nevertheless, phytotoxic effects have also been described for Ag-NPs [116]. In studies on callus cultures of *Populus nigra* L., small 7–9 nm bifunctionalized silver nanoparticles (Ag-NPs-Cit-L-Cys) and silver nitrate were evaluated. The NPs significantly reduced callus FW, induced oxidative stress, and were more toxic than AgNO_3_. A significant increase in FW and DW of embryogenic wheat callus cells was observed after treatment with Ag-NPs (3 or 4 mg L^−1^), Cu-NPs (0.015 or 0.02 mg L^−1^), and their combination compared with conventional analogues, AgNO_3_ and CuSO_4_ (0.025 mg L^−1^) [121]. Optimal regeneration was achieved with the combined application of 4 mg L^−1^ Ag-NPs and 0.015 mg L^−1^ Cu-NPs, which increased regeneration by 21%. The effectiveness of alloyed AgAu-NPs (25–35 nm) was also demonstrated in the callus culture of *Prunella vulgaris* L., where their addition to the medium increased protein, phenol, and flavonoid synthesis [117].

It is well established that iron nanoparticles can function as fertilizers and enhance plant metabolism by increasing the activity of enzymes involved in respiration and photosynthesis [122]. In vitro callus culture of black cumin (*Nigella sativa* L.), widely known for its anticancer, anticoronaviral, and antibacterial properties due to its rich content of bioactive secondary metabolites, represents a promising approach for producing these valuable ones. To stimulate callus growth and secondary metabolite biosynthesis in *N. sativa*, FeO_3_-CTs NPs (50–100 nm), synthesized by co-precipitation with chitosan, were added to MS medium containing 2 mg L^−1^ 2,4-D and 1 mg L^−1^ BA. The NPs were applied at four concentrations (0, 50, 100, and 200 mg L^−1^) under different LED light spectra and in darkness: darkness (D), white light (W, 450–640 nm), blue light (B, 450 nm), and red light (R, 640 nm), at an intensity of 2000 lx [102].

After 40 days of cultivation, the highest FW, DW, phenolic, flavonoid, and amino acid contents were observed under red light in combination with 100 mg L^−1^ FeO_3_-CTs NPs. Concentrations of 100 and 200 mg L^−1^ had the most pronounced effects on secondary metabolite production. DPPH antioxidant activity and carbohydrate accumulation increased after treatment with 200 mg L^−1^ FeO_3_-CTs in darkness. In addition, treatment with 100 mg L^−1^ more effectively stimulated secondary metabolite production under blue light. These results demonstrate a strong dependence of NP effects on the light spectrum.

A synergistic role of Fe_3_O_4_-NPs in combination with plant hormones in the induction of organogenesis and enhancement of secondary metabolite accumulation has also been shown, emphasizing their potential in medicinal plant biotechnology. In callus culture of *Hypericum perforatum* L., treatments were performed using different concentrations of Kn (0.5, 1.0, and 1.5 mg L^−1^), 2,4-D (0.5, 1.0, and 2.0 mg L^−1^), and Fe_3_O_4_-NPs (1, 2, and 4 mg L^−1^) [113]. The optimal treatment consisted of MS medium with 0.5 mg L^−1^ 2,4-D, 2 mg L^−1^ Kn, and 4 mg L^−1^ Fe_3_O_4_-NPs. Under these conditions, plant height and root length increased; callus FW, shoot number, and regeneration efficiency (51%) increased threefold compared with the control without hormonal supplementation. In addition, high levels of hypericin, hyperforin, β-patchoulene, and hexadecanol were detected, accounting for 93% of the total peak area, and total phenolic and flavonoid contents increased by 80%.

MnO-NPs 10–40 nm in size, synthesized by a green method using *Syzygium cumini* (L.) Skeels leaf extract, exerted a stimulatory effect on callus growth and biomass accumulation in *Moringa oleifera* Lam. At a concentration of 10 mg L^−1^, while also preventing contamination [118]. These results highlight the considerable potential of manganese-containing NPs for further investigation. However, to date, this remains the only study devoted to the application of such NPs.

Promising results have also been reported for TiO_2_-NPs with an average size of 24.5 nm in callus cultures of *Hordeum vulgare* L. [119]. The highest tested concentration, 60 mg L^−1^, provided the most pronounced callus induction and biomass increase, suggesting the possibility of even greater effectiveness at higher concentrations and requiring further study. No negative changes in callus quality were observed under the treatments. Biosynthesized TiO_2_-NPs may also function as elicitors in plant systems. The effects of physically synthesized perlite NPs and biosynthesized TiO_2_/perlite nanocomposites were evaluated in callus cultures of *Hypericum perforatum* L. [31]. The synthesized perlite nanoparticles (14–23 nm) and TiO_2_/perlite nanoparticles (15–24 nm) affected the volatile compound profile of *H. perforatum*. The tested concentration range of 25–200 mg L^−1^ did not exert phytotoxic effects on callus growth.

Se-NPs (40–100 nm), biosynthesized using *Allium sativum* L. extract, were applied at different concentrations (0, 50, 100, 200, and 400 µg L^−1^) to assess their effects on callus proliferation and secondary metabolite biosynthesis under different light regimes: standard illumination of 2000–2500 lx, diffuse light of 500–1000 lx, and complete darkness, followed by transfer of explants to a full-light regime [120]. Maximum FW accumulation, phenolic and flavonoid contents, and antioxidant activity were observed after treatment with 0.1–0.2 mg L^−1^ Se-NPs in darkness for two weeks.

**Figure 2:**
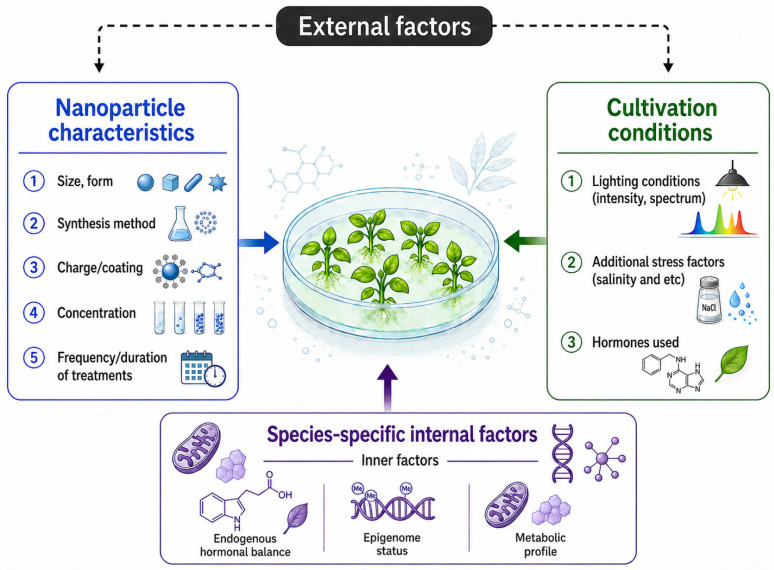
Factors influencing the efficiency of callus elicitation by NPs.

Available studies show that the choice of NPs and optimization of their application conditions for callus elicitation should be performed individually. Potential elicitation strategies depend on multiple factors (Figure 2), including internal factors, such as the metabolic characteristics of the cultured species, and external factors, such as NP properties, including size, synthesis method, surface coating, and others, as well as cultivation conditions, including light regime, applied phytohormones, additional elicitors, and related parameters.

Successful integration of all the factors mentioned above may make the use of NPs as elicitors for stimulating the synthesis of target metabolites in callus cultures substantially more effective and may potentially increase production efficiency several-fold.

Similarly, when NPs are used to stimulate secondary metabolism, three types of endpoints should be distinguished: biomass accumulation, the concentration of the target compound in the tissue, and the total metabolite yield per unit of biomass. These parameters may change in different directions: stress-induced elicitation can increase the concentration of the target compound while simultaneously reducing biomass accumulation, which does not always result in an increased total product yield. Therefore, increased antioxidant enzyme activity, elevated levels of ROS-dependent markers, or higher phenolic compound content should be considered indicators of defense metabolism induction, but not evidence of technological efficiency. For cultures intended for food, cosmetic, or pharmaceutical applications, data on the accumulation of NPs or released ions in the biomass, their possible transfer into extracts, and their residual content in the culture medium are also important.

### 3.2. Hairy Root Culture

Hairy root culture represents another modern biotechnological approach for the large-scale in vitro production of secondary metabolites from biochemically active and genetically stable root systems transformed by *Rhizobium rhizogenes* (formerly *Agrobacterium rhizogenes*) [123]. This soil bacterium penetrates plant cells through wounded tissues, recognizing them via specific phenolic compounds released by the plant in response to damage. Upon contact with plant cells, virulence genes located on the root-inducing (Ri) plasmid are activated, enabling the transfer of a DNA fragment (T-DNA) from the bacterium into the plant cell nucleus. Genes within the T-DNA region (*rolA*, *rolB*, and *rolC*) play a key role in initiating uncontrolled root proliferation [124]. These genes alter the cellular hormonal balance, primarily through auxin-mediated pathways, disrupting growth regulation and triggering rapid cell division and the formation of thin, highly branched “hairy” roots.

Inoculation can be performed both in vitro and in planta. Currently, two fundamentally distinct methods are used to establish in vitro hairy root cultures: (1) direct plant inoculation, in which plant explants are introduced into a liquid culture of *R. rhizogenes*, and (2) co-cultivation of plant explants with a bacterial suspension [125,126]. After transformation, hairy roots grow rapidly without the addition of phytohormones, maintain genetic and biochemical stability, and often synthesize target compounds at levels comparable to or exceeding those of intact plants. These cultures are widely used for the production of pharmacologically important compounds, especially when whole-plant cultivation is difficult or inefficient [124].

Hairy root cultures retain tissue specificity and physiological characteristics typical of underground organs. One of the key objectives of current research is to develop strategies and optimize conditions to further improve the efficiency of hairy root culture systems [127]. Both biotic and abiotic elicitors may be used to stimulate the synthesis of target compounds by activating plant immune responses and modulating the expression of genes involved in secondary metabolite biosynthetic pathways [128]. As promising abiotic stimulators, NPs are able to increase the growth rate and metabolite production in hairy root cultures of various plant species [129].

For example, the application of TiO_2_-NPs (22–30 nm) in hairy root culture of *Saponaria officinalis* L. resulted in a threefold increase in FW, as well as elevated total phenolic and flavonoid contents (Table 5) [126]. It was shown that hairy root growth was affected not only by NP concentration but also by treatment duration and frequency (24 and 48 h). However, the effect on polyphenol concentration was nonlinear and showed no clear pattern.

Treatment of *Brassica rapa* var. *rapa* with Ag-NPs (2–10 nm) increased the levels of gallic acid, quercetin, and flavonols, probably through the induction of oxidative stress, as confirmed by increased malondialdehyde (MDA) and H_2_O_2_ levels [130].

In hairy root cultures of *Hyoscyamus reticulatus* L. and *Hyoscyamus pusillus* L., presoaking explants in SiO_2_-NP solutions (10–100 nm) at concentrations of 25 and 100 mg L^−1^ produced a pronounced stimulatory effect that depended on both dose and exposure time [32]. In *H. reticulatus*, treatment with 25 mg L^−1^ increased FW and hyoscyamine accumulation and also enhanced scopolamine content, especially after 48 h of soaking. At the higher concentration of 100 mg L^−1^ combined with 24 h of soaking, increases in FW and accumulation of both alkaloids were also observed.

In *H. pusillus*, treatment at 25 mg L^−1^ with a 48 h exposure resulted in a twofold increase in biomass, higher levels of phenolic compounds, and enhanced accumulation of scopolamine and hyoscyamine. It has also been shown that hairy roots of *Hybanthus enneaspermus* (L.) F. Muell. Accumulate selenium considerably more efficiently than intact in vitro plants, with the highest uptake of Se particles observed at a concentration of 50 mg L^−1^ [131]. Although natural roots are also capable of selenium accumulation [132], this process is substantially more efficient in hairy root cultures.

At the same time, studies on *Panax ginseng* C.A. Mey. Showed that supplementation with 0.5 mmol selenium increased both saponin content and productivity in hairy root cultures by 1.3-fold compared with the control [133]. In *Cichorium intybus* L., it was shown that NP supplementation affected biomass accumulation: Se-NPs (5 mg L^−1^) increased biomass to 6.7 g, whereas Ag-NPs (5 mg L^−1^) suppressed growth by more than twofold relative to the control [134]. In hairy root cultures, the application of AuCu-NPs also appears promising, since they have been shown to stimulate growth and secondary metabolism in adventitious root cultures of *S. rebaudiana* [59].

**Table 5:** Application of NPs in hairy root culture.

NPs	Size, nm	Obtaining Method	Plant Culture	Explant	Concentration, mg∙L^−1^	Experimental Conditions	Experiment Results	Reported Adverse Effects/Additional Information	Ref.
TiO_2_	22–30	Chemical synthesis	*Saponaria officinalis* L.	roots	10	MS	↑ FW (3 times) when 48 h treatment, ↑ TPC (+75%) 24 h treatment, ↑ antioxidant capacity (+38%) when 48 h treatment, ↑ APX (+50%) when 48 h treatment, ↑ protein SO6 production (+76.5%) ↑ rosmarinic acid (+23.4%)	No obvious toxicity was indicated at 10 mg L^−1^. At 20 mg L^−1^: ↓ FW to 0.96 g (−68.9%) No obvious toxicity was indicated at 50 mg L^−1^	[135]
					50		↑ TPC (2.5 times) 48 h treatment, ↑ antioxidant capacity (2 times), ↑ protein SO6 production (2.1 times), ↑ antioxidant capacity (+81%) when 24 h treatment, ↑ CAT (2 times) when 24 h treatment, ↑ APX (2 times) when 48 h treatment		
Ag	2–10	Biological synthesis using *Bacillus marisflavi*	*Brassica rapa* var. *rapa*	hairy roots	100	MS	↑ gallic acid (+22%), ↑ quercetin (+15%), ↑ MDA (2.5 times), ↑ H_2_O_2_ (+38%), ↑ glucosinolates: glucoallysin, glucobrasicanapine, sinigrin, progoitrin, gluconapine, 4-methoxyglucobrasicin, 4-hydroxyglucobrasicin, glucobrasicin, neoglucobrasicin, and gluconasturtiin (+2–6%) and their transcripts (MYB34, MYB51, MYB28 and MYB29), ↑ flavonols (+15%), ↑ hydroxybenzoic acid (+16%), ↑ hydroxycinnamic acid (+19%), ↑ TPC and TFD, and their transcripts at 48 h on the 23rd day of cultivation	↑ MDA, 2.5-fold, and ↑ H_2_O_2_ (+38%), indicating induction of oxidative stress	[130]
SiO_2_	10–100	Chemical synthesis	*Hyoscyamus reticulatus* L.	hairy roots from callus of cotyledon explants	25	24 or 48 h of soaking MS	↑ FW (2 times), ↑ hyoscyamine (1.8 times) when 48 h treatment, ↑ scopolamine (+24%) when 24 h treatment, (2 times) when 48 h treatment	No explicit toxic effects were indicated at 25 mg L^−1^	[32]
					100	24-h treatment on day 21 of cultivation MS	↑ scopolamine (3.6 times), ↑ hyoscyamine (13 times)	No explicit toxic effects were indicated at 100 mg L^−1^/24 h	
			*Hyoscyamus pusillus* L.	Hairy roots from leaf callus	25	48-h soaking on day 21 of cultivation MS	↑ FW (2 times), ↑ TPC (+40%), ↑ scopolamine (2 times), ↑ hyoscyamine (1.5 times)	No adverse effects were indicated	

The table includes only adverse effects, control treatments, genetic stability data, and other safety-related observations that were explicitly reported in the original sources. If ionic controls, residual amounts of NPs/metals, genetic stability, abnormal morphology, acclimatization survival, or long-term growth parameters are not indicated for a particular study, this means that these parameters were not reported in the corresponding source. Abbreviations: ↑—Increase in indicators, ↓—Decrease in indicators, FW—fresh weight, DW—Dry weight, MS—Murashige and Skoog medium, TFD—total flavonoid, TPC—total phenolics content, APX—Ascorbate peroxidase, CAT—Catalase, PAL—phenylalanine ammonia-lyase, MDA—Malondialdehyde, PPO—Polyphenol oxidase.

In addition to the NPs described above, MgO-, S-, Fe_2_O_3_-, Mn-, Mo-, and Ca-NPs can also be considered promising for application in tissue culture. These materials have demonstrated effectiveness as fertilizers or fertilizer components [14]. Their application should be carefully coordinated with the concentrations of the corresponding bulk elemental forms in the culture medium to prevent potential adverse effects associated with cumulative elemental excess. Furthermore, broader investigation of Se-NPs as medium supplements appears promising, given their wide range of biological activities [136], including the activation of growth processes and stimulation of secondary metabolite biosynthesis.

## 4. Application Effectiveness of Nanoparticles In Vitro Depending on Their Properties and the Plant Class for Which They Are Used

The efficiency of NPs evidently depends both on their intrinsic properties (composition and geometry) and on the target object (the taxonomic affiliation of the plant). Importantly, NPs may be effective with respect to certain callus characteristics while being ineffective or even exerting the opposite effect on others. For example, they may enhance shoot regeneration efficiency while reducing anthocyanin concentration [20]. Thus, NP efficiency can be assessed using different criteria.

### 4.1. Dependence of NP Efficiency on Composition, Size, and Concentration

Manuscripts for quantitative analysis were selected using the same principles as for compiling Table 1, Table 2, Table 3, Table 4 and Table 5. The search was conducted using combinations of keywords: “Plant” + “Tissue Culture” + “Nanoparticles”, “Plant” + “Micropropagation” + “Nanoparticles”, “Plant” + “Multiplication” + “Nanoparticles”, “Callus” + “Nanoparticles” or “in vitro rooting” + “Nanoparticles”. The search of manuscripts and its full text was carried out in scientific databases PubMed (https://pubmed.ncbi.nlm.nih.gov/, access date 10 April 2026), Google Scholar (https://scholar.google.com/, access date 15 April 2026), MDPI web site (https://www.mdpi.com/, access date 21 April 2026), ResearchGate (https://www.researchgate.net/, access date 21 April 2026). The search period spanned the years 2000–2026. Preference was given to manuscripts published in 2020 or later. Manuscripts published in high-ranking Q1 and Q2 journals according to Web of Science and/or Scopus were reviewed first. However, for innovative results or rare studies, papers were reviewed regardless of the journal’s ranking.

Before conducting a generalized analysis, we adopted the following condition: each quantitative value reported in the literature published in a single study was obtained through a parallel, independent measurement and can be analyzed outside the scope of a specific study. Thus, we can combine data from different studies and disaggregate data from a single study if necessary. This assumption is based on important properties of the analyzed data. First, a single study measures several parameters affecting completely different aspects of plant physiology and morphology, and there cannot be a quantitative correlation between the results published in a single study. Therefore, generalizing all experimental results based on the “one study—one data point” principle will lead to an unjustified reduction in information and uncertainty regarding the classification of the resulting “chimeric data point.” Second, the nature of the data has a greater impact on their quantitative values than the experimental conditions. For example, gene expression varies by 2–3 orders of magnitude, protein expression and metabolite concentrations by no more than 1 order of magnitude, and morphological parameters can vary by units or tens of percent. Therefore, combining data from a single study is impractical. However, gene expression data or plant morphometric characteristics from different studies vary similarly, which facilitates analysis. Third, effects within a single data set can be multidirectional: for example, the activity of enzyme 1 may decrease, while the activity of enzyme 2 increases. Averaging the activities of enzymes 1 and 2 will result in a loss of information. However, isolating data on the activity of enzyme 1 from different studies allows for an adequate assessment of the influence of NP properties on this parameter. Thus, each averaged quantitative values reported in the literature was analyzed as an independent value (data point on each of the graphs). The detailed process of quantitative analysis is described above.

At the first stage, we evaluated the dependence of NP efficiency on their composition, size, and concentration, as well as on combinations of these parameters. To enable comparison among effects, we calculated a dimensionless effect size modulus using Equation (1):

(1) $$Effect\;modulus={log}_{10}\left(\left|\left(\frac{A-∆A}{A}\right)*100\%\right|\right)$$

where A is the initial value of the measured parameter (number of shoots, chlorophyll concentration, enzyme activity, metabolite content, etc.), and ΔA is the value of the same parameter after NP treatment (expressed in the same units as A).

To assess trends in the relationships between size and effect modulus, size and effective concentration, and effective concentration and effect modulus, linear regression lines were constructed and r^2^ values were calculated. To evaluate the combined effects of size, composition, and/or concentration, 3D maps were generated using Kriging correlation method [137]. The statistical significance of the effective parameter ranges identified in the 3D maps, as compared to random point distributions, was assessed by calculating the z-score using Equation (2):

(2) $$z_{i}=\left(x_{i}-µ\right)/\sigma$$

where zi is the z-score of the individual value i, x is the individual value i, μ is the mean, and σ is the standard deviation. Differences were considered statistically significant at zi ≥ 2.

In assessing the dependence of NP efficiency on composition, we included only those NP types described in at least two studies covered in this review. The following classification of NPs, based on the analyzed publications, was adopted: Ag (pristine and modified), Au (pure gold and gold-based composites), Cu (Cu and CuO), Fe_2_O_3_ (pristine and modified), ZnO, C (including MWCNTs and modified graphene oxide), Se, and Si (including SiO_2_).

**Figure 3:**
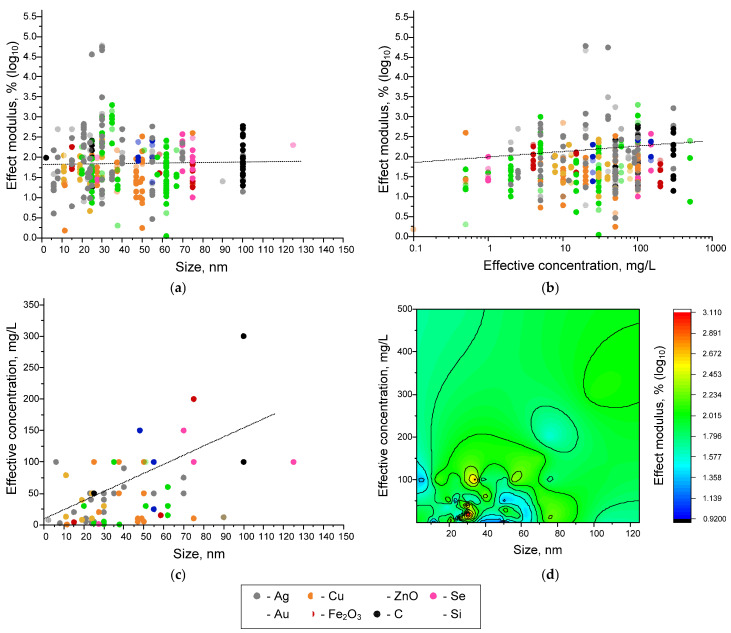
Assessment of the relationships between size and efficacy (**a**), effective concentration and efficacy (**b**), size and effective concentration (**c**), and all three parameters combined (**d**) for all analyzed NPs. Data points represent individual published results. In total, 508 quantitative values reported in the literature were analyzed. When experimental conditions coincided, the corresponding points overlap but are indicated by more saturated colors. Dashed lines represent the results of linear regression analysis. The yellow, orange, and red regions on the 3D plot (3D) indicate areas where zi ≥ 2. In panels (**a**–**c**), colors denote the principal chemical elements comprising the nanoparticles: Ag (light gray), Au (yellow), Cu (orange), Fe (red), Zn (green), carbon (dark gray), Se (pink), and Si (blue).

To evaluate the overall contribution of NP size and concentration, nanoparticles of all compositions were combined into a generalized group (Figure 3).

No direct dependence of the effectiveness (effect modulus) of the generalized NP group on their concentration was observed (Figure 3a). The trend line is nearly horizontal (r^2^ < 0.01). Notably, the ranges of effective sizes of NPs with different compositions substantially overlap. Slightly smaller sizes can be distinguished for Ag-NPs compared to ZnO or Se NPs; however, the effectiveness of all NPs is comparable. Therefore, NP size and effectiveness show only weak dependence on their composition. Carbon-based NPs may be considered separately, although this is likely attributable to specific features of their structure and synthesis.

No clear relationship between NP effectiveness and concentration was identified either (Figure 3b). The trend suggests a slight increase in effectiveness with increasing NP concentration; however, the correlation is negligible (r^2^ = 0.02). No significant differences were found among NPs of different compositions in terms of the distribution of effective concentrations.

A more pronounced trend was observed when analyzing the relationship between NP size and concentration: a decrease in size was associated with lower effective concentrations (Figure 3c). Overall, this tendency is consistent with the concept that reducing NP size increases their active surface area (and, consequently, their effectiveness) [138]. Nevertheless, this relationship can be characterized as very weak (r^2^ = 0.2). In several cases, analyzing the combined influence of two parameters on a third provides more insight than pairwise comparisons. In particular, this approach has proven effective in evaluating the biological effects of physical factors, including mechanical, acoustic, and light exposure [137,139,140].

Analysis of the relationship between NP size, concentration, and efficacy revealed the presence of clearly localized “windows” or ranges of sizes and concentrations in which NPs exhibit maximal effectiveness (Figure 3d). This phenomenon is frequently observed when analyzing the responses of biological systems to combinations of parameters. According to the constructed 3D map, in the pooled dataset, the most pronounced responses were more frequently observed at NP sizes of approximately 20–60 nm and concentrations of approximately 1–120 mg L^−1^, corresponding to the yellow, orange, and red regions of the 3D map. However, these ranges should be interpreted as areas in which pronounced responses are overrepresented in published studies rather than as universal optimal parameters. They may reflect not only biological patterns but also the distribution of experimental designs used, the choice of model objects, and publication bias. These regions are characterized by z_i_ > 2, indicating a statistically significant deviation from a random distribution.

Thus, our analysis shows that the majority of studies report a relatively narrow range of optimal concentrations and sizes for generalized NPs. The boundaries of this range correspond to the fundamental limits described in the literature. NPs smaller than 10 nm are considered capable of autonomous cellular penetration [138,141]. NPs in the size range of 10–100 nm exert their effects primarily through receptor-mediated mechanisms, whereas particles larger than 100 nm typically demonstrate activity only under specific conditions [141]. This raises a reasonable question: is this range universal for all NPs regardless of composition, or does it represent an “averaging” effect of distinct optimal ranges specific to individual NP types?

To address this question, we analyzed the above-described relationships separately for each NP type. The largest number of studies included in our analysis focused on Ag-NPs and ZnO-NPs; therefore, these will be discussed in greater detail.

**Figure 4:**
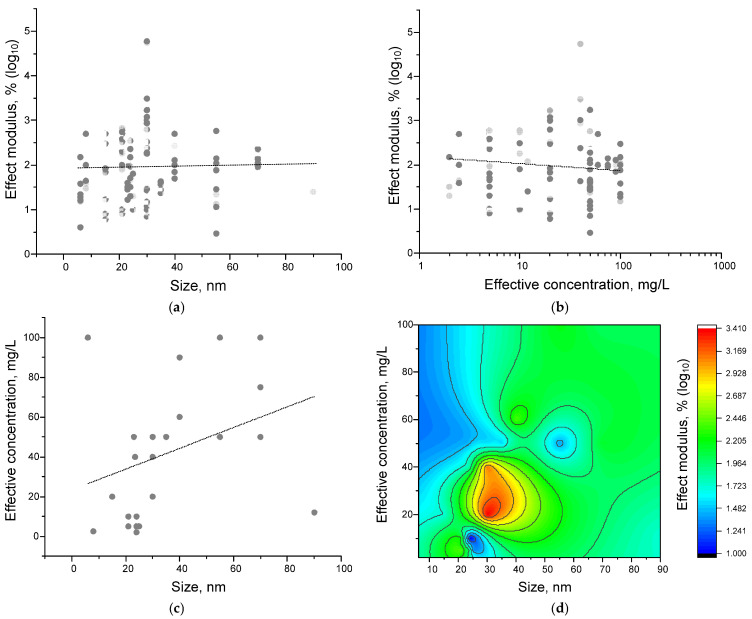
Assessment of the relationship between particle size and efficacy (**a**), effective concentration and efficacy (**b**), size and effective concentration (**c**), and all three parameters combined (**d**) for Ag-based nanoparticles. Points represent individual published results. In total, 127 quantitative values reported in the literature were analyzed. In cases where experimental conditions overlapped, the points coincide but are indicated with more saturated colors. Dashed lines represent the results of linear regression analysis. Orange and red regions on the 3D map indicate areas with z-scores ≥ 2.

For Ag-NPs, a pattern similar to that observed for the generalized group was identified (Figure 4). The magnitude of the biological effects of Ag-NPs showed virtually no dependence on either size or concentration (r^2^ < 0.01 in both cases) (Figure 4a,b). A positive trend between effective concentration and particle size was observed, consistent with the generalized analysis (Figure 4c); however, this relationship did not reach statistical significance (r^2^ = 0.09). The 3D mapping revealed a region of maximal efficacy within the size range of approximately 25–40 nm and concentration range of 15–45 mg L^−1^ (Figure 4d).

**Figure 5:**
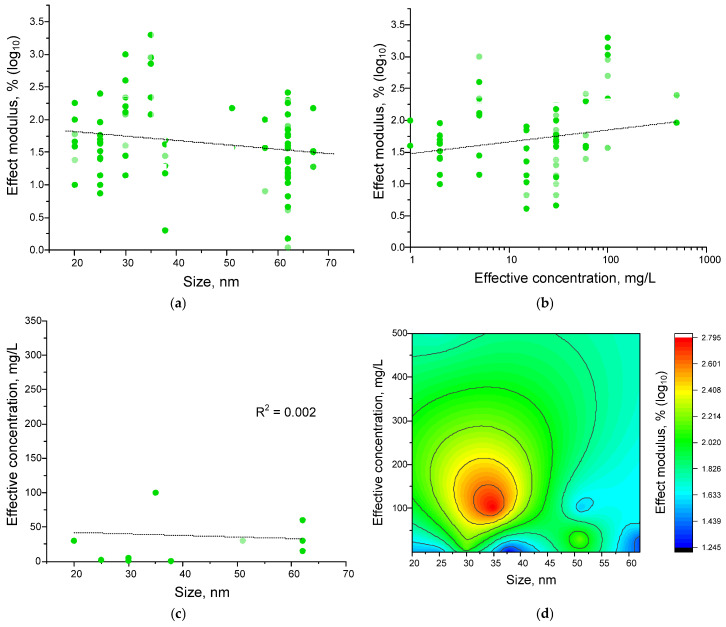
Assessment of the relationship between NPs size and efficacy (**a**), effective concentration and efficacy (**b**), size and effective concentration (**c**), and all three parameters combined (**d**) for ZnO-based NPs. Points represent individual published results. A total of 120 quantitative values reported in the literature were analyzed. When experimental conditions coincided, the points overlap but are indicated with more saturated colors. Dashed lines represent the results of linear regression analysis. Orange and red regions on the 3D map indicate areas with z-scores ≥ 2.

For ZnO nanoparticles (ZnO-NPs), trends toward increased efficacy with decreasing particle size and increasing concentration were observed (Figure 5a–c). However, these trends did not reach statistical significance (r^2^ = 0.02–0.05). The 3D map clearly reveals a “window” of maximum efficacy at particle sizes of approximately 20–60 nm and concentrations of 75–200 mg L^−1^ (Figure 5d). Thus, the efficacy patterns of Ag-NPs and ZnO-NPs may vary depending on composition, yet they are consistent with the results obtained for the aggregated dataset.

**Figure 6:**
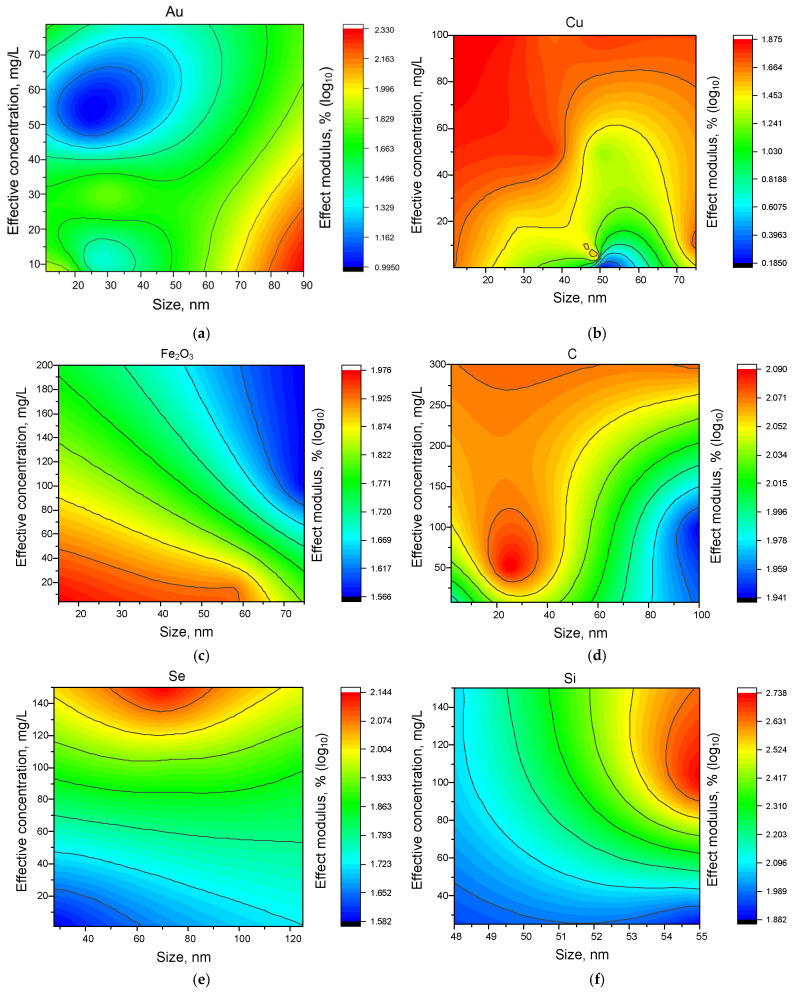
Evaluation of the relationship between NP size, effective concentration, and efficacy for Au- (**a**), Cu- (**b**), Fe_2_O_3_- (**c**), C- (**d**), Se- (**e**), and Si-based (**f**) NPs. The numbers of quantitative values analyzed were 36, 75, 32, 56, 26, and 13, respectively. Orange and red regions on the 3D maps indicate areas with z-scores ≥ 2.

For clarity, only 3D maps will be provided for all remaining types of NPs. The generated 3D maps for each type of NP reveal regions of high and low efficacy (Figure 6). For Se and Si NPs, the “windows” of maximum efficacy are narrowly localized within size and concentration ranges of 50–80 nm and 120–150 mg L^−1^ (Se-NPs, Figure 6e), and 54–55 nm and 90–145 mg L^−1^ (Si-NPs, Figure 6f). In contrast, Au, Cu, C, and Fe_2_O_3_ NPs exhibit broader and/or more complex ranges of effective sizes and concentrations.

Au-NPs demonstrate the highest efficacy within the size range of 70–90 nm and concentrations of 5–40 mg L^−1^ (Figure 6a). However, additional regions at 10–20 nm and 25–35 nm within the same concentration range (5–40 mg L^−1^) also show slightly elevated efficacy. Notably, no data are available for Au NPs smaller than 60 nm at concentrations above 35 mg L^−1^. It is possible that Au NPs exhibited toxic effects under these conditions.

Cu-NPs show high efficacy at sizes of 20–65 nm and concentrations above 60 mg L^−1^ (Figure 6b). Interestingly, Cu-NPs with diameters of 50–60 nm appear to be the least effective. Fe_2_O_3_-NPs demonstrate high efficacy across a broad size range of 10–65 nm, but within a concentration range of 5–80 mg L^−1^ (Figure 6c). C-NPs exhibit high efficacy at sizes of 15–40 nm and concentrations ranging from 25 to 300 mg L^−1^ (Figure 6d). Overall, the behavior of C- and Fe_2_O_3_-NPs is consistent with the principle of increased efficacy with decreasing particle size. However, in specific cases, C-NPs larger than 90 nm may also be effective at concentrations exceeding 290 mg L^−1^.

Despite the individual characteristics of the 3D “size–concentration–efficacy” maps for NPs of different compositions, their most effective size and concentration ranges fall within the broader group interval of 10–100 nm and 50–200 mg L^−1^. Thus, despite the presence of distinct windows of pronounced response, NP size and concentration did not show a strong universal linear relationship with effect magnitude in the pooled dataset. Therefore, further comparison of NPs by composition should be considered a generalized analysis limited by the heterogeneity of the source data and does not eliminate the need for stage-specific optimization of size and concentration for particular cultures, plant species, and endpoints.

**Figure 7:**
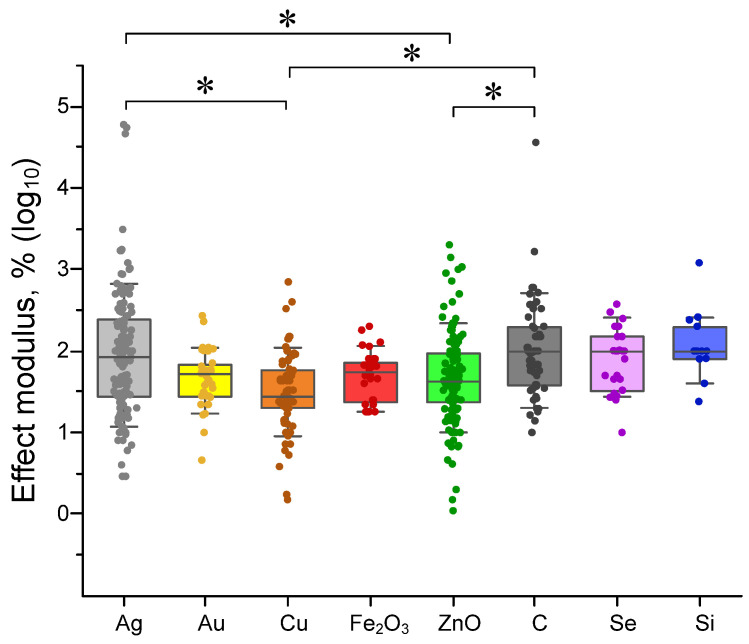
Dependence of the overall efficiency of NPs on their composition. Data are presented as medians and the 10th, 25th, 75th, and 90th percentiles. Each point corresponds to an individual analyzed published quantitative value. *—*p* < 0.05, Kruskal–Wallis one-way analysis of variance on ranks with post hoc Dunn’s method.

An analysis of the overall efficacy of NPs with different compositions revealed that Ag- and C-NPs were generally more effective than ZnO- and Cu-NPs (Figure 7).

### 4.2. Dependence of NP Efficiency on the Botanical Affiliation of Plants

**Figure 8:**
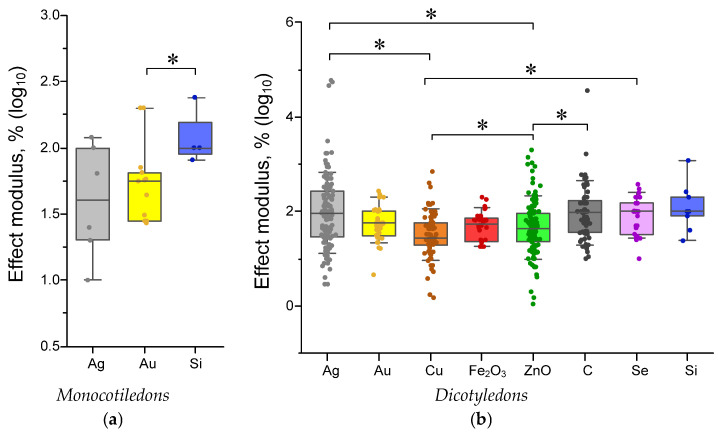
Dependence of the overall efficiency of NPs on plant taxonomic affiliation: *Monocotyledons* (**a**) and *Dicotyledons* (**b**). Data are presented as medians and the 10th, 25th, 75th, and 90th percentiles. Each point corresponds to an individual analyzed published quantitative value. *—*p* < 0.05, Mann–Whitney test (**a**); Kruskal–Wallis one-way analysis of variance on ranks with post hoc Dunn’s method (**b**).

At the next stage, we examined the extent to which plant taxonomy and functional characteristics determine NP efficiency. As the first parameter, we selected systematic classification: monocotyledonous versus dicotyledonous plants. The results of the analysis are presented below (Figure 8). It should be noted that *Dicotyledons* are used as research objects much more frequently than *Monocotyledons*. Among the analyzed studies, data for monocots were available only for three types of NPs—Ag, Au, and Si (Figure 8a). According to the analysis, Si NPs were somewhat more effective than Au-NPs.

For dicots, studies were conducted for all analyzed NP types (Figure 8b). In this case, more statistically significant differences were identified than in the generalized comparison (Figure 7 and Figure 8). Ag-NPs were more effective, when considering the trend rather than the overall effect, than Cu- and ZnO-NPs. Se-NPs were also more effective than Cu-NPs. C-NPs demonstrated higher efficacy than ZnO-NPs. It should be noted that species- and cultivar-specific plant responses [28,32,86,87] contribute to the variability of the observed positive effects.

### 4.3. Efficiency of NPs in Relation to Different Functional Responses

At the final stage, we assessed whether NPs of different compositions differ in their efficiency with respect to various plant functions. The main evaluated functions included:

(a) Accumulation of metabolites (proteins, phenolics, flavonoids, scopolamine, etc.).
(b) Antioxidant activity (activities of SOD, catalase, DPPH, etc.).
(c) Growth rate of callus cells and whole plants.

**Figure 9:**
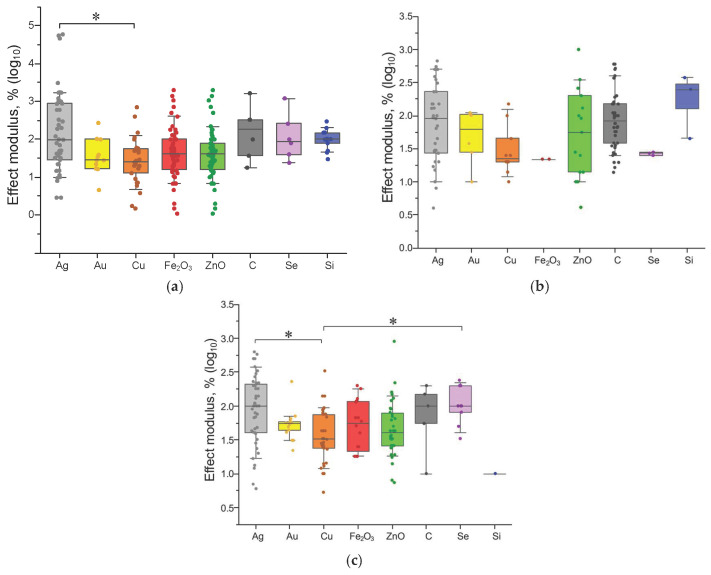
Dependence of metabolite synthesis efficiency (**a**), antioxidant defense (**b**), and growth rate (**c**) on NP composition. Data are presented as medians with the 10th, 25th, 75th, and 90th percentiles. Each point represents an individual analyzed published quantitative value. *—*p* < 0.05, Kruskal–Wallis one-way analysis of variance on ranks with post hoc Dunn’s method.

When evaluating the efficiency of primary and secondary target metabolite production, Ag-NPs were found to be the most effective, whereas Cu-NPs were the least effective (Figure 9a). In contrast, when assessing the effects of NPs on antioxidant activity, NPs of all compositions exhibited comparable effects on plant antioxidant defense (Figure 9b). At the same time, this result characterizes the magnitude of published responses but does not necessarily reflect the total product yield, since metabolite concentration, biomass accumulation, and overall culture productivity may change in different directions.

Metabolite synthesis proved to be the most responsive plant parameter to nanoparticles of different compositions (Figure 9). Ag- NPs and Se-NPs most effectively stimulated the production of target metabolites, including primary metabolites (proteins, sugars) and secondary metabolites (phenolics, flavonoids, essential oils, etc.). Cu-NPs stimulated metabolite synthesis significantly less effectively than Se-NPs. Thus, Ag- NPs and Se-NPs can be considered the most effective for enhancing plant metabolism. The wide range of effective concentrations indicates variable plant responses to elicitation depending on species and cultivation conditions.

## 5. Safety and Methodological Limitations

Despite the promising effects of NPs in plant in vitro culture, their application should be regarded as stage-specific and dependent on the endpoint being assessed rather than as a universal approach to growth stimulation. In culture systems, NPs may perform different functions: they may act as antimicrobial agents, sources of nutrients or released ions, modulators of stress responses, factors of ROS-mediated signaling, elicitors of secondary metabolism, or, at excessive concentrations, toxic agents that disrupt growth, morphogenesis, and genetic stability [11,12]. After addition to the nutrient medium, NPs may aggregate or agglomerate, dissolve with ion release, interact with agar, salts, organic compounds, plant growth regulators, and exudates, and adsorb onto the surface of plant tissues [64]. These processes may substantially alter NP availability, biological activity, and the final response of the culture.

For metallic and metal oxide NPs, the observed effects may be caused not only by the NP form itself but also by ion release, surface reactivity, ROS generation, and physical interactions with cell walls or membranes. Since metal ions play independent roles in biological systems [142], the use of ionic controls is particularly important when interpreting such data. Thus, in several studies, the effects of Se-NPs were compared with selenate or bulk Se, which made it possible to distinguish between the effects of NP and ionic/bulk forms of selenium [66,67]. For Ag-NPs, it has also been shown that their effects may differ substantially from those of AgNO_3_: in *P. nigra* callus culture, small bifunctionalized Ag-NPs reduced callus FW, induced oxidative stress, and exhibited more pronounced toxicity than AgNO_3_ [116]. By contrast, in wheat embryogenic callus, Ag-NPs, Cu-NPs, and their combinations were compared with the bulk analogues AgNO_3_ and CuSO_4_, which allowed a more accurate assessment of the contribution of the NP form to callus growth and regeneration [121]. Consequently, without appropriate ionic controls, such as AgNO_3_, CuSO_4_, CuCl_2_, ZnSO_4_, or iron salts, it is difficult to determine unambiguously whether the observed response is nanoparticle-specific or predominantly ion-mediated.

Safety assessment is especially important in studies where NPs are used to obtain planting material or biomass intended for food, cosmetic, pharmaceutical, or agricultural applications. For several cultures, it has been shown that NPs may be taken up by plant tissues and accumulate in them. For example, in *S. rebaudiana* treated with Ag-NPs, silver content in tissues increased in a dose-dependent manner, and the NPs themselves were detected in stem epidermal cells, vascular bundles, intercellular spaces, leaf veins, and stomata [56]. In shoot culture of *P. armeniaca*, Ag accumulation also depended on the cultivation system and NP concentration, with higher silver content in shoots in the temporary immersion system than on semi-solid medium [86]. In *O. petraeum*, TEM confirmed the uptake and translocation of Ag- and Cu-NPs in tissues, while in *S. fruticosa*, EDX analysis showed the presence of Se-NPs in tissues or on the shoot surface after treatment [85,91]. These data indicate the need to assess residual levels of NPs or corresponding metals in regenerants, cultivated biomass, extracts, and spent media.

Adverse effects of NPs may also manifest as phytotoxicity, oxidative stress, growth inhibition, disruption of morphogenesis, or reduced genetic stability. For example, in *R. lutea*, increasing the ZnO-NP concentration to 60 mg L^−1^ was accompanied by reduced shoot biomass, decreased chlorophyll, protein, phenolic, and flavonoid contents, and increased TBARS, proline, and antioxidant enzyme activity, indicating the development of a stress response [82]. In *A. multiflora* culture, CuO-NP concentrations above 20 mg L^−1^ induced oxidative stress and phytotoxicity, while 40 mg L^−1^ completely inhibited rooting [84]. In experiments with *S. rebaudiana*, high concentrations of CuO- and ZnO-NPs reduced rooting, biomass, antioxidant activity, and steviol glycoside accumulation [60]. In *M. acuminata*, Ag-NPs stimulated growth parameters but simultaneously increased lipid peroxidation [88]. These examples show that increased antioxidant enzyme activity, proline content, or ROS-related markers should not always be interpreted as a positive effect; in some cases, such changes reflect NP-induced stress.

Genetic stability of regenerated plants and cell cultures requires particular attention. In *Chrysanthemum × grandiflorum* culture, Ag-NPs reduced the multiplication factor and increased the proportion of polymorphic plants to 28.2–32%, indicating a possible reduction in the genetic stability of regenerants [87]. In *R. nervosus* callus, treatment with Ag-NPs was accompanied by 12.5% polymorphism, although the culture remained relatively stable [115]. At the same time, some studies confirmed genetic stability, for example in *R. lutea* after ZnO-NP treatment using flow cytometry [82]. Therefore, for micropropagation protocols, especially in commercial production of planting material, it is necessary to assess not only the multiplication factor and shoot growth but also the frequency of abnormal morphogenesis, vitrification, somaclonal variation, acclimatization survival, and long-term plant productivity.

The quantitative comparison performed in this review also has methodological limitations. Effect magnitude allows the standardized comparison of the scale of published responses; however, it combines heterogeneous endpoints, including shoot number, biomass, chlorophyll content, enzyme activity, nutrient uptake, and metabolite accumulation. These parameters reflect different biological processes and should not be considered equivalent indicators of practical efficiency. Therefore, the identified NP size and concentration ranges should be regarded as literature-derived trends and intervals for formulating working hypotheses rather than as universal recommendations. Further development of this field requires studies with complete dose–response series, ionic controls, assessment of residual amounts, analysis of genetic stability, regenerant quality, biomass safety, and possible release of threatening NPs or metal ions into the environment [143].

## 6. Conclusions

Analysis of published studies showed that high concentrations of NPs exert toxic effects on plants through the generation of ROS, to which plants respond by activating both enzymatic and non-enzymatic antioxidant defense mechanisms. One important manifestation of this protective response is the enhanced biosynthesis of metabolites, many of which have commercial value. Therefore, identifying optimal NP concentrations capable of triggering defense responses without reducing biomass growth, or even while stimulating it, is of particular importance. However, such responses should be interpreted with regard to the endpoint being assessed, since increased antioxidant activity or metabolite accumulation is not always accompanied by improved biomass accumulation, regenerant quality, or total yield of the target product.

NPs have diverse chemical forms, ranging from amphiphilic molecules to metal oxides and from synthetic polymers to biomolecules. Their synthesis methods, size, and surface charge confer unique properties that determine their application features and effectiveness. In plant in vitro tissue culture, ZnO-, CuO-, and Ag-NPs have been studied most extensively, whereas TiO_2_-, SiO_2_-, and Se-NPs have received comparatively less attention despite their considerable applied potential. However, the effects of NPs depend not only on their composition and physicochemical characteristics but also on the culture stage, plant species and genotype, explant type, nutrient medium composition, exposure duration, and the endpoint selected for evaluating effectiveness.

A generalized analysis shows that, for plants cultured in vitro, the most effective NP size range is 20–60 nm at concentrations of 1–120 mg L^−1^. These values should be regarded as literature-derived trends in the analyzed dataset rather than as universal optimal conditions for all NPs, plant species, and culture systems. Specifically, for the most extensively studied Ag-NPs and ZnO-NPs in plant tissue culture, the effective size ranges are 25–40 nm and 15–45 nm, respectively, while the effective concentrations are 15–45 mg L^−1^ and 75–200 mg L^−1^. Cu-NPs are more effective at sizes of 20–65 nm and concentrations above 60 mg L^−1^, whereas Fe_2_O_3_-NPs are effective across a broad size range of 10–65 nm at concentrations of 5–80 mg L^−1^. Au-NPs show the highest effectiveness at sizes of 70–90 nm and concentrations of 5–40 mg L^−1^. For Se-NPs, the effective ranges are 50–80 nm and 120–150 mg L^−1^, and for Si-NPs, 54–55 nm and 90–145 mg L^−1^. Au-, Cu-, C-, and Fe_2_O_3_-NPs demonstrate more complex and broader ranges of effective sizes and concentrations. This variability indicates the need for further dose-dependent testing of NPs separately for micropropagation, rooting, callus growth, hairy root cultures, and metabolite production.

Comparative analysis of overall NP effectiveness by composition shows that, in the analyzed dataset and according to pooled endpoints, Ag- and C-NPs are more effective than ZnO- and Cu-NPs. However, this ranking should not be extrapolated to all stages of in vitro culture, since the same NP type may stimulate one endpoint while simultaneously inhibiting another. For maximizing the production of target metabolites, including primary metabolites such as proteins and sugars and secondary metabolites such as phenolic compounds, flavonoids, essential oils, and others, Ag- and Se-NPs appear to be the most suitable, whereas Cu-NPs are less effective. At the same time, in metabolite production systems, biomass accumulation, target compound concentration, and total product yield must be considered separately, since an increase in metabolite concentration does not always indicate improved production efficiency of the culture.

The practical application of NPs also requires safety assessment. For metallic and metal oxide NPs, appropriate ionic controls must be used. In addition, when developing protocols for micropropagation and biomass production, it is necessary to consider possible phytotoxicity, oxidative stress, abnormal morphogenesis, somaclonal variation, genetic instability, accumulation of NPs or metals in tissues, transfer of residual amounts into extracts, as well as issues related to disposal of spent media and potential release into the environment.

Nanoparticles have substantial but still insufficiently explored potential for application in plant in vitro culture. Currently, there are no studies addressing the stability of NPs in agarized media during autoclaving, which is particularly important for non-metallic NPs and low-stability colloidal NP solutions. NP further use should be based on stage-specific optimization, standardized assessment of positive and negative effects, analysis of residual amounts, and verification of the quality of regenerated plants or the biomass obtained. This field is expected to develop rapidly in the coming years and to become particularly prominent in agricultural biotechnology.

## Figures and Tables

**Table 1:** The use of NPs on the plant introduction into in vitro culture stage (sterilization and shoot initiation).

NPs	Size, nm	Obtaining Method	Plant	Explant	Concentration, mg∙L^−1^	Experimental Conditions	Experiment Results	Reported Adverse Effects/Additional Information	Ref.
AgO	40–100	Chemical synthesis by hydrothermal method	*Kaempferia parviflora* Wall. ex Baker	Rhizome buds	100	Immersion of rhizome buds for 60 min before introduction into tissue culture MS + 8 mol L^−1^ BA + 0.2 TDZ	↓ contamination without affecting the survival of explants (100%)	NaOCl alone did not ensure complete decontamination; no reduction in survival was observed under the optimal AgO-NP treatment	[19]
CuO	50	Chemical synthesis (Sigma-Aldrich, St. Louis, MO, USA)	*Betula pubescens* Ehrh.	Apical micropropagated shoots	0.1	MS	↑ number of viable explants (+5.3%)	No reduction in explant viability was observed at low CuO-NP concentrations	[65]
					5		↓ development of colonies of phytopathogens *A. alternata* and *F. avenaceum* (−68.42%) without reducing the viability of explants, ↑ expression of stress resistance genes (MYB46, LEA8, PAL)	The antifungal effect depended on the pathogen species; suppression of *F. oxysporum* was limited or absent	
Se	10–45	Chemical synthesis	*Momordica charantia* L.	Seeds	1	MS	↑ leaf FW (+50%), stem FW (+100%), root FW (+33%), ↑ NR (+28%), ↑ APX (+27%) in roots, ↑ PAL in roots (+25%); ↑ diameter of the stem, bark layer and central cylinder, ↑ stimulation of the development of primary and secondary tissues	Selenate/bulk Se control included; Se-NPs at ≥10 mg L^−1^ caused toxic effects, including stem bending and impaired root meristem development	[66]
Se	50–200	Biological synthesis using *Lactobacillus acidophilus*	*Nicotiana tabacum* L. cv. Ottawa	Segments 1 cm long	100	MS	↑ FW (3 time), ↓ total chlorophyll (−27%), there are no significant changes in the structure and functions of the photosynthetic apparatus (unlike selenate)	↓ total chlorophyll content (−27%); selenate control included	[67]
Graphene oxide (GO)	1.9–2.5	Green synthesis using oil palm fruit clusters *Elaeis guineensis* Jacq.	*Fragaria* × *ananassa*	Shoot tips	7.5	MS + 8 mmol TDZ	Stimulation of seedling development, genetic similarity of more than 97%	Genetic similarity of regenerated plants was >97% according to molecular marker analysis	[68]
MWCNT	20–30	Chemical synthesis	*Catharanthus roseus* (L.) G.Don	Seedlings	50	MS	↑ leaf surface area (2 times), ↑ FW (2.5 times), ↑ leaf FW (3 times), ↑ root length (+55%), ↑ total protein (+37%), ↑ CAT (2.7 times), POD (2 times), PAL (+37%), ↑ TPC (+18%), ↑ alkaloids (2 times) ↑ DAT gene transcription (37 times), ↑ the size of cells and conductive tissues of the xylem, stimulation of callus initiation	↑ callus initiation; this response may be useful for callus cultures but undesirable for direct micropropagation	[29]
α-Fe_2_O_3_	38–79	Green synthesis using extract of *Cymbopogon jwarancusa* (Jones) Schult. leaves	*Cicer arietinum* L.	Embryo axes with the root apex removed, callus	15	MS + 1.5 mg L^−1^ 2,4-D	↑ regeneration frequency from 40 to 88%, ↑ shoot length (+40%), ↑ root initiation (+83%)	NP treatment enhanced regeneration in a recalcitrant crop	[69]

The table includes only adverse effects, control treatments, genetic stability data, and other safety-related observations that were explicitly reported in the original sources. If ionic controls, residual amounts of NPs/metals, genetic stability, abnormal morphology, acclimatization survival, or long-term growth parameters are not indicated for a particular study, this means that these parameters were not reported in the corresponding source. Abbreviations: ↑, increase/stimulation; ↓, decrease/inhibition; MS, Murashige and Skoog medium; BA, 6-benzyladenine; TDZ, thidiazuron; GO, graphene oxide; MWCNT, multi-walled carbon nanotubes; CAT, catalase; POD, peroxidase; PAL, phenylalanine ammonia-lyase; APX, ascorbate peroxidase; TPC, total phenolic content.

## Data Availability

No new data were created or analyzed in this study.

## Abbreviations

The following abbreviations are used in this manuscript:

NPs	nanoparticles
FW	Fresh weight
DW	Dry weight
MS	Murashige and Skoog nutrient medium
MF	Multiplication factor
TFD	Total flavonoid
TPC	Total phenolics content
NR	Nitrate reductase
APX	Ascorbate peroxidase
CAT	Catalase
POD	Peroxidase
SOD	Superoxide dismutase
PAL	phenylalanine ammonia-lyase
GSH	Glutathione
GR	Glutathione reductase
GPX	Glutathione peroxidase
MDA	Malondialdehyde
ROS	Reactive oxygen species
GA3	Gibberellic acid
IAA	Indole-3-acetic acid
NAA	1-Naphthaleneacetic acid
DPPH	1,1-diphenyl-2-picrylhydrazyl
TBARS	Thiobarbituric acid reactive substances
ABA	Abscisic acid
IBA	Indole-3-butyric acid
2iP	N6-(delta-2-isopentenyl)adenine
FRAP	Ferric Reducing Antioxidant Power
PPO	Polyphenol oxidase
TDZ	Thidiazuron
BA	6-Benzylaminopurine
2,4-D	2,4-Dichlorophenoxyacetic acid
TIS	Temporary immersion system
LED	Light-emitting diode
